# YAP inhibition overcomes adaptive resistance in HER2-positive gastric cancer treated with trastuzumab via the AKT/mTOR and ERK/mTOR axis

**DOI:** 10.1007/s10120-024-01508-3

**Published:** 2024-05-23

**Authors:** Jiao Qiao, Mei Feng, Wenyuan Zhou, Yuan Tan, Shuo Yang, Qi Liu, Qingchen Wang, Weimin Feng, Yisheng Pan, Liyan Cui

**Affiliations:** 1 Institute of Medical Technology, Peking University Health Science Center, Beijing, 100191 China; 2 Department of Laboratory Medicine, Peking University Third Hospital, Beijing, 100191 China; 3 Core Unit of National Clinical Research Center for Laboratory Medicine, Peking University Third Hospital, Beijing, 100191 China; 4 Translational Cancer Research Center, Peking University First Hospital, Beijing, 100034 China; 5 Division of General Surgery, Peking University First Hospital, Peking University, No. 8 Xi Shiku Street, Beijing, 100034 China; 6 Key Laboratory of Carcinogenesis and Translational Research (Ministry of Education/Beijing), NMPA Key Laboratory for Research and Evaluation of Radiopharmaceuticals (National Medical Products Administration), Department of Nuclear Medicine, Peking University Cancer Hospital & Institute, Beijing, 100142 China

**Keywords:** HER2-positive gastric cancer, Trastuzumab, Adaptive resistance, YAP, Drug combination

## Abstract

**Background:**

Human epidermal growth factor receptor 2 (HER2)-positive gastric cancer (GC) is a heterogeneous GC subtype characterized by the overexpression of HER2. To date, few specific targeted therapies have demonstrated durable efficacy in HER2-positive GC patients, with resistance to trastuzumab typically emerging within 1 year. However, the mechanisms of resistance to trastuzumab remain incompletely understood, presenting a significant challenge to clinical practice.

**Methods:**

In this study, we integrated genetic screening and bulk transcriptome and epigenomic profiling to define the mechanisms mediating adaptive resistance to HER2 inhibitors and identify potential effective therapeutic strategies for treating HER2-positive GCs.

**Results:**

We revealed a potential association between adaptive resistance to trastuzumab in HER2-positive GC and the expression of YES-associated protein (YAP). Notably, our investigation revealed that long-term administration of trastuzumab triggers extensive chromatin remodeling and initiates YAP gene transcription in HER2-positive cells characterized by the initial inhibition and subsequent reactivation. Furthermore, treatment of HER2-positive GC cells and cell line-derived xenografts (CDX) models with YAP inhibitors in combination with trastuzumab was found to induce synergistic effects through the AKT/mTOR and ERK/mTOR pathways.

**Conclusion:**

These findings underscore the pivotal role of reactivated YAP and mTOR signaling pathways in the development of adaptive resistance to trastuzumab and may serve as a promising joint target to overcome resistance to trastuzumab.

**Supplementary Information:**

The online version contains supplementary material available at 10.1007/s10120-024-01508-3.

## Introduction

The epidermal growth factor receptor family (ErbB family) consists of several members, including erbB1 (EGFR, HER1), erbB2 (also known as HER2), erbB3 (HER3), and erbB4 (HER4) [1–3]. HER2, encoded by the ERBB2 gene on chromosome 17, acts as a proto-oncogene. It is a 185 kD transmembrane protein, also known as p185, featuring tyrosine kinase activity but lacking endogenous ligands [4, 5]. Notably, it can form heterodimers with other ERBB family members, activating diverse signaling pathways, such as the PI3K, MAPK, and JAK/STAT pathways, to promote cell proliferation and suppress cell apoptosis [6–10]. HER2 was first found to be functionally associated with human breast cancer pathogenesis in 1987. HER2-positive breast cancer is linked to a greater risk of local growth and distant metastasis [11]. Furthermore, HER2 is found in various cancer types, especially prevalent in GC, where approximately 10–15% of GC patients exhibit HER2 overexpression or gene amplification [12].

Trastuzumab was approved in 2006 for the adjuvant treatment of HER2-positive node-positive breast cancer and in 2010 for the treatment of HER2-positive metastatic stomach cancer. The ToGA (trastuzumab for GC) clinical trial established the use of trastuzumab in combination with chemotherapy as the first-line treatment for HER2-positive advanced GC [13]. In this pivotal study, the combination of trastuzumab and chemotherapy achieved a median overall survival (OS) of 13.8 months, with an impressive objective response rate (ORR) of 47%. However, despite the initial success of trastuzumab in treating HER2-positive GC, most patients stopped responding to trastuzumab within 1 year, and acquired resistance limited the duration of the trastuzumab response. Overcoming trastuzumab resistance remains a crucial challenge in the management of HER2-positive GC [14]. At present, a substantial body of research on the molecular mechanisms underlying resistance to trastuzumab predominantly originates from investigations into breast cancer. Widely acknowledged mechanisms involve PTEN and INPP4B deficiency [15–17]. The prevalence of PTEN mutations exhibits subtype-specific variations within breast cancer, the frequency is approximately 5% in the HER2-positive subtype and notably higher at 35% in the triple-negative subtype [18, 19]. In addition, factors such as HER2 heterogeneity, loss of HER2-positive status, acquired HER2 mutations, HER2 heterodimerization, altered intracellular signaling, and the tumor immune microenvironment all contribute to HER2 drug resistance [14, 20]. Nevertheless, limited preclinical investigations and clinical trials of trastuzumab have been performed on GC patients, and thus a comprehensive inspection of the pharmacological effect of trastuzumab in GC is lacking. Therefore, there is an urgent need to comprehensively characterize the mechanism of trastuzumab resistance and explore innovative combinations of new drugs to overcome trastuzumab resistance.

Yes-associated protein (YAP), a downstream effector molecule of the Hippo signaling pathway, acts as a transcriptional coactivator and has been identified in numerous human malignancies, including GC [21–23]. Additionally, recent studies have proposed that YAP is a mediator influencing pancreatic cancer chemosensitivity [24]. Cao et al. reported that increased YAP status can serve as a predictive factor for improved survival rates in breast cancer patients [25]. Shi et al. found that the HER4–YAP1 axis potentially governs the acquired resistance of HER2-positive GC cells to trastuzumab by promoting epithelial–mesenchymal transition, thereby conferring enhanced proliferative capacity upon HER2-positive GC cells exposed to trastuzumab [26]. However, the link between YAP and trastuzumab resistance in HER2-positive GC cells remains unclear. Therefore, in this study, we conducted a comprehensive genome-wide CRISPR screen to identify YAP and investigated whether YAP inhibition affects the efficacy of trastuzumab in HER2-positive GC cells. We aimed to investigate the possible mechanism of the development of trastuzumab resistance to improve the therapeutic effect of trastuzumab in patients with HER2-positive GC.

## Materials and methods

Further information is available in the Supplementary Methods.

## Results

### Genome-wide CRISPR screening identified YAP as a key gene for anti-HER2 resistance in GC

**Fig. 1:**
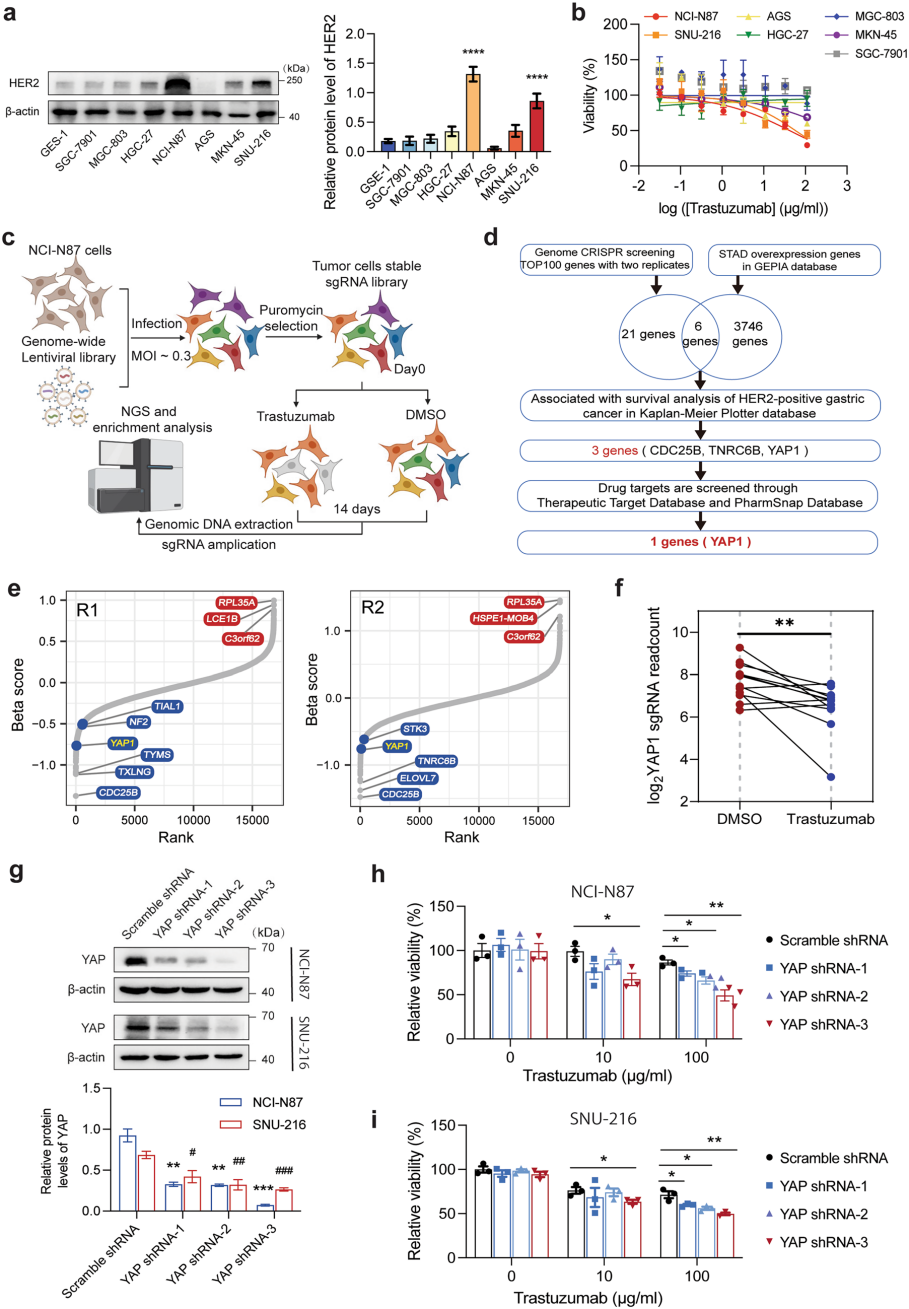
Identification of trastuzumab sensitizers in HER2-positive GC cells. **a** The expression of HER2 in 7 GC cell lines and normal GES-1 gastric mucosa epithelium cell lines quantified by Western blotting. **b** The viability of seven GC cell lines was detected by CCK-8 assays after trastuzumab (0–100 μg/ml) treatment for 72 h was detected by CCK-8 assay. **c** Schematic capture of the genome-wide CRISPR screening of NCI-N87 cells. The schematic was created with BioRender.com with permission. **d** Bioinformatics analysis revealed YAP1 as a possible combined target for trastuzumab resistance. **e** Rank of gene essentiality according to the genome-wide CRISPR screen in NCI-N87 cells in the trastuzumab and vehicle groups, and the differentially expressed genes were sorted by MAGeCKFlute. The data are presented as two replicates. **f** The sgRNAs targeting YAP1 were depleted in trastuzumab-treated NCI-N87 cells. **g** Western blot analysis revealed that YAP was knocked down after shRNA transfection in NCI-N87 (upper) and SNU-216 (lower) cells. **P < 0.01 and ***P < 0.001 versus scramble shRNA in NCI-N87 cells; ^#^*P* < 0.05, ^##^*P* < 0.01, and ^###^*P* < 0.001 versus scramble shRNA in SNU-216 cells. **h**, **i** The relative viability of NCI-N87 (left) and SNU-216 (right) cells in the presence of 10 μg/ml or 100 μg/ml trastuzumab for 3 d versus vehicle. Cells were pretreated with YAP shRNA or scramble shRNA. The data are presented as the mean ± SEM of three replicates. *P < 0.05, **P < 0.01 and ***P < 0.001 versus scramble shRNA

Some GC tissues exhibit dysregulation of HER2 (Fig. S1a), specifically notably upregulation of HER2 expression compared to that in adjacent normal gastric tissues (Fig. S1b). Additionally, HER2 expression was significantly correlated with survival outcomes in patients with GC (Fig. S1c). To gain a more comprehensive understanding of the pharmacological effects of trastuzumab resistance and identify the optimal preclinical model for drug screening in combination therapy for GC, we utilized western blotting to identify HER2-positive GC cell lines, namely, NCI-N87 and SNU-216, among seven GC cell lines (Fig. 1a). Next, the effectiveness and specificity of trastuzumab were assessed. The administration of trastuzumab reduced in the proliferation of HER2-positive GC cells (NCI-N87 and SNU-216 cells). In contrast, other HER2-negative cells exhibited resistance to trastuzumab (Fig. 1b). Thus, we selected NCI-N87 cells for genome-wide CRISPR knockout (KO) screening to systematically explore potential synergistic targets for trastuzumab (Fig. 1c). During the 2-week screening, sgRNA-targeting genes critical for viability, referred to as resistance-related genes, were depleted under trastuzumab treatment. The experiment was repeated with high reproducibility (Fig. S1d). Several genes known to be related to resistance in HER2-positive cases, such as CDK12 [27], CTNNB1 [28], and FOXM1 [29], were identified, confirming the reliability of our screening method (Tables S2, S3). Next, we intersected the top 100 genes from genome-wide CRISPR screening with upregulated genes in STAD from the GEPIA database, and 6 common genes were identified. Among them, three genes were associated with the survival of patients with HER2-positive GC, and the target gene (*YAP1*) was identified as a drug target by the Therapeutic Target Database and PharmSnap Database (Fig. 1d). Therefore, we focused on YAP as a potential combination target (Fig. 1e). Upon trastuzumab treatment of NCI-N87 cells, three out of six sgRNAs targeting YAP1 decreased YAP expression in one replicate CRISPR screening, while the other three sgRNAs decreased YAP expression in both sequencing runs (Fig. 1f). Pan-cancer analysis revealed that HER2 and YAP1 are highly essential genes (gene effect score < -1) for GC (Fig. S1e, f). YAP was found to be upregulated in GC (Fig. S1g, h) and was correlated with a poor prognosis in HER2-positive GC patients (Fig. S1i) [30, 31]. Additionally, components of the mTORC1 signaling pathway, including late endosomal/lysosomal adaptor, MAPK, and MTOR activator 1 (LAMTOR1) and late endosomal/lysosomal adaptor, MAPK and MTOR activator 5 (LAMTOR5), assumed greater significance in the presence of trastuzumab, with their loss overcoming trastuzumab resistance (Tables S2, S3). Consistent with the CRISPR screening results, YAP knockdown had a minimal impact on the proliferation of NCI-N87 and SNU-216 cells. However, the combination of YAP knockdown and trastuzumab treatment significantly supppressed cell proliferation (Fig. 1g–i).

### Synergistic effect of trastuzumab and verteporfin in HER2-positive GC cells

**Fig. 2:**
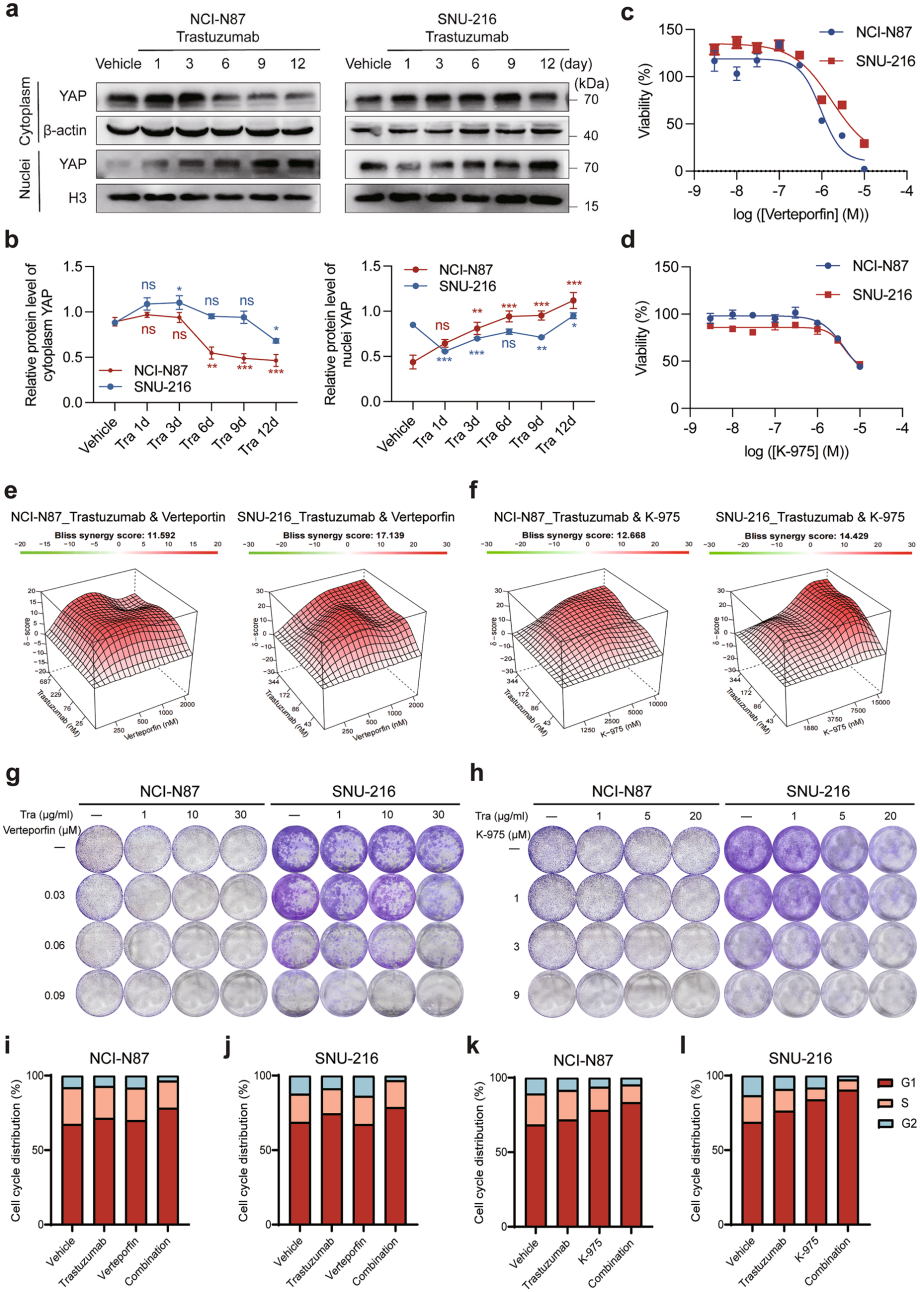
Cellular effects of the combination of trastuzumab and a YAP inhibitor in HER2-positive GC cells. **a** Western blot analysis of cytoplasmic and nuclear YAP protein levels in NCI-N87 (left) and SNU-216 (right) cells in the treatment with trastuzumab at the indicated time points. **b** Quantification of the protein levels of cytoplasmic and nuclear YAP protein levels in NCI-N87 (left) and SNU-216 (right) cells after trastuzumab treatment at the indicated time points using ImageJ software. The data are presented as the mean ± SEM of three replicates. *P < 0.05, **P < 0.01 and ***P < 0.001 versus vehicle. **c**, **d** The viability of NCI-N87 and SNU-216 cells was detected by CCK-8 assays after verteporfin (0–10 μM, upper) or K-975 (0–10 μM, lower) treatment for 72 h. **e** Synergistic model and scores of trastuzumab and verteporfin in NCI-N87 (left) and SNU-216 (right) cells. **f** Synergistic model and scores of trastuzumab and K-975 in NCI-N87 (left) and SNU-216 (right) cells. **g** NCI-N87 and SNU-216 cells were treated with trastuzumab (1, 10, 30 μg/ml), verteporfin (0.03, 0.06, 0.09 μM), or a combination of trastuzumab and verteporfin for 2 weeks, after which colony crystal violet staining was performed. **h** NCI-N87 and SNU-216 cells were treated with trastuzumab (1, 10, 30 μg/ml), K-975 (1, 3, 9 μM), or a combination of trastuzumab and K-975 combine in pairs for colony formation assays. **i-l** Representative histograms of the G1, S, and G2 phases of NCI-N87 cells treated with vehicle, trastuzumab (30 μg/ml), verteporfin (1 μM), K-975 (3 μM), or the combination of both

To explore the process of adaptive resistance to trastuzumab, we treated NCI-N87 and SNU-216 cells with infigratinib for various durations spanning from 1 to 12 days and analyzed YAP expression in the cytoplasm and nucleus at each time point, observing notable changes over the time course. Specifically, as the duration of trastuzumab treatment increased, nuclear YAP (N-YAP) levels significantly increased, accompanied by a decrease in cytoplasmic YAP levels, indicating enhanced YAP translocation into the nucleus (Fig. 2a, b). Subsequently, we conducted the following combination therapy assay using two YAP inhibitors. Currently, verteporfin, which targets the pivotal YAP gene within the HIPPO pathway, is being studied in clinical studies primarily for choroidal neovascularization and macular degeneration, while preclinical investigations have been initiated for K-975. To evaluate the sensitivity of HER2-positive cells to YAP inhibition, we performed dose–response tests on NCI-N87 and SNU-216 cells treated with verteporfin or K-975. Both verteporfin and K-975 exhibited anti-proliferative effects on both cell lines, with IC50 value of 0.96 μM for verteporfin and 4.31 μM for K-975 in NCI-N87 cells. For SNU-216 cells, the IC50 value were 1.72 μM for verteporfin and 4.18 μM for K-975 (Fig. 2c, d, Fig. S2a).

We then assessed the efficacy of combining the YAP inhibitor (verteporfin or K-975) with trastuzumab in inhibiting HER2-positive cell proliferation. Bliss collaborative analysis revealed a synergistic effect between trastuzumab and verteporfin (or K-975) in NCI-N87 and SNU-216 cells (Fig. 2e, f, Fig. S2b, c). Furthermore, colony formation experiments demonstrated that treatment with trastuzumab combined with verteporfin or K-975 combination treatment inhibited the growth of NCI-N87 and SNU-216 cells (Fig. 2g, h).

We next assessed the cell cycle distribution of NCI-N87 cells after 48 h of treatment with vehicle, trastuzumab, verteporfin (or K-975), or the combination (Fig. 2i–l, Fig. S2d–g). While trastuzumab and verteporfin (or K-975) alone allowed normal cell cycle progression to the S phase comparable to that in untreated cells, their combination resulted in cell cycle arrest at the G1/S phase, causing a delayed entry into the S phase. The results indicated that YAP inhibitors sensitize cells to cell cycle arrest, potentially contributing to the slower cell proliferation observed. Taken together, these results provide important clinical insight, suggesting that the combination of trastuzumab and YAP inhibitors leads to growth inhibition and cell cycle arrest, resulting in superior anti-tumor effects.

### PI3K/mTOR signaling feedback drives adaptive resistance to trastuzumab

**Fig. 3:**
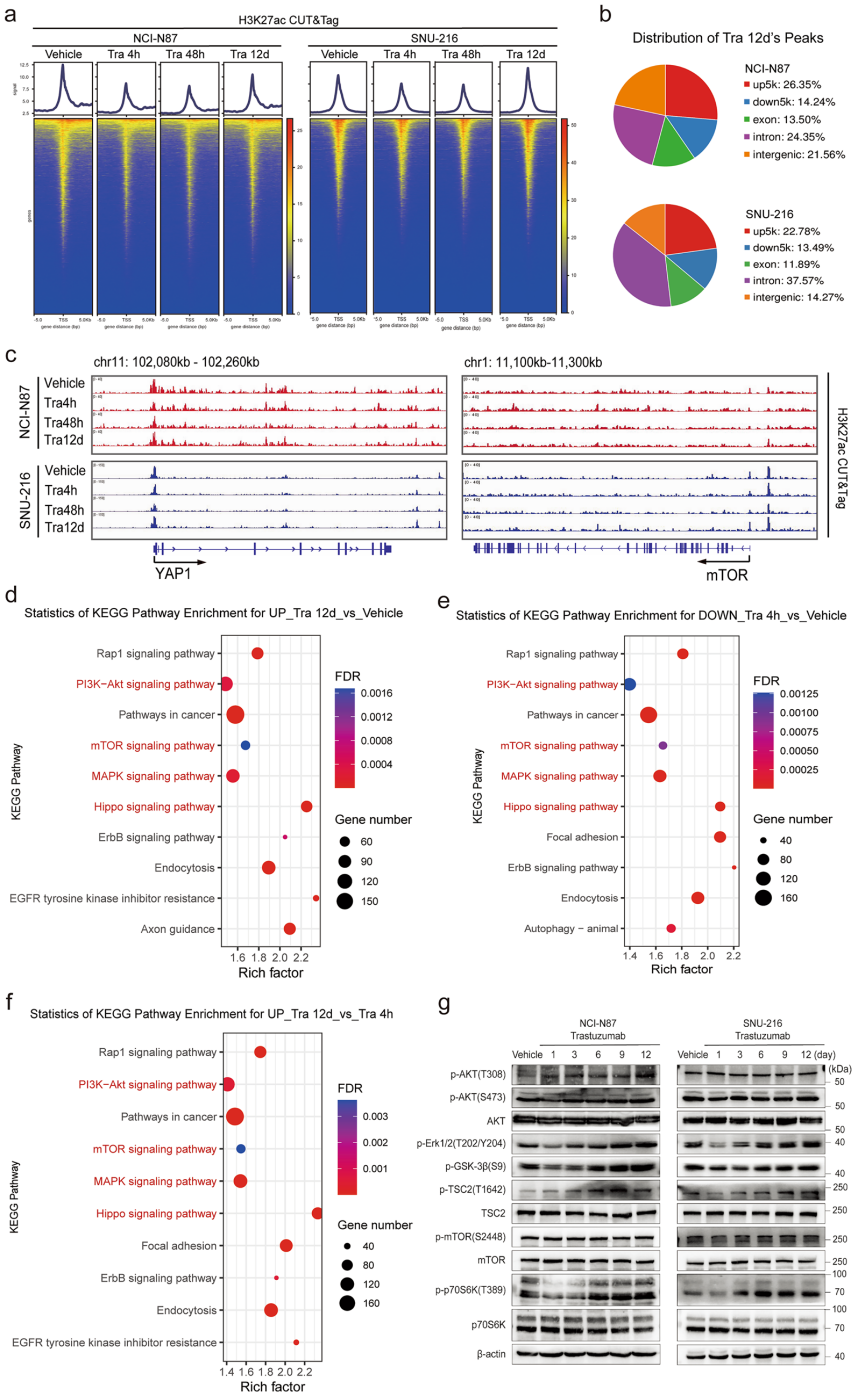
YAP re-activation and mTOR pathway feedback drive adaptive resistance to trastuzumab in HER2-positive GC cells. **a** TSS heatmap showing that the H3K27ac signal was significantly decreased after short-term trastuzumab treatment for 4 h, but increased from 48 h to 12 days. **b** Gene tracks depicting H3K27ac signals at the YAP (left) and mTOR (right) loci. The signal from trastuzumab at the indicated time points was normalized to that of the vehicle at the same time points. **c** Pie plot of the genomic distribution of H3K27ac peaks in NCI-N87 cells treated with trastuzumab for 12 days. **d**–**f** The common genes with increased H3K27ac peaks in NCI-N87 and SNU-216 cells with trastuzumab versus Vehicle for 12 days (**d**), decreased H3K27ac peaks with trastuzumab versus vehicle for 4 h (**e**), the increased H3K27ac peaks with trastuzumab treatment for 12 days versus 4 h (**f**) were subjected to KEGG pathway enrichment analysis. **g** Western blot analysis of AKT, ERK1/2, GSK-3β, TSC2, mTOR, and p70S6K phosphorylation and total AKT, TSC2, mTOR, and p70S6K protein levels in NCI-N87 (upper) and SNU-216 (lower) cells treated with trastuzumab (30 μg/ml) at the indicated time points

To identify the regulatory mechanisms that contribute to the adaptive resistance upon trastuzumab inhibition, we examined changes in chromatin accessibility over a time course of trastuzumab treatment in NCI-N87 cells by sequencing (ATAC-seq) (Fig. S3a–d). To further determine whether the trastuzumab-induced adaptive resistance process is driven by chromatin changes, we performed cleavage under targets and tagmentation (CUT&Tag) sequencing of H3K27ac in NCI-N87 and SNU-216 cells. Consistent with chromatin accessibility, the H3K27ac signal was reduced after a short-term trastuzumab treatment for 4 h. Nevertheless, it was subsequently reactivated over the course of the following 12 days (Fig. 3a). Notably, YAP and mTOR signaling-related genes displayed similar patterns during trastuzumab adaptive resistance, with initial inhibition followed by reactivation of YAP transcription (Fig. 3b, Fig. S3e). Transcriptional enhanced associate domain (TEAD)-mediated transcriptional regulation is the primary signaling event downstream of YAP. We found that the TEAD family (TEAD1/4) and YAP exhibited similar H3K27ac modifications (Fig. S3f). In addition, 45.91% of the peaks in NCI-N87 cells treated with trastuzumab for 12 days were located within distal intergenic regions (21.56%) or introns (24.35%), while 51.84% of the peaks in SNU-216 cells treated with trastuzumab for 12 days were located within distal intergenic regions (14.27%) or introns (37.57%) (Fig. 3c). To analyze the expression characteristics of common differentially expressed genes in trastuzumab treated NCI-N87 and SNU-216 cells, KEGG pathway enrichment analyses were conducted. We observed activation of Hippo signaling as well as the upregulation of PI3K-AKT, MAPK, and mTOR signaling pathways treated with trastuzumab for 12 days compared to vehicle (Fig. 3d). In addition, during adaptive resistance to trastuzumab, the aforementioned signaling pathways associated with H3K27ac-enriched regions were initially subjected to transient inhibition, followed by subsequent reactivation (Fig. 3e, f). These results demonstrate that treatment of HER2-driven GC with trastuzumab activates a feedback loop leading to AKT/mTOR and ERK/mTOR activation, which promoted cell growth and survival, thereby decreasing trastuzumab inhibitor efficacy.

To further investigate the adaptive resistance to trastuzumab, we treated NCI-N87 and SNU-216 cells with trastuzumab for 1, 3, 6, 9, and 12 days and analyzed YAP and mTOR signaling at each time point. In agreement with our ATAC-seq and CUT&Tag data, short-term trastuzumab treatment (1–3 days) decreased the nuclear expression of YAP in SNU-216 cells and significantly increased nuclear expression after prolonged trastuzumab inhibition (Fig. 2a). In the first 1–3 days of trastuzumab treatment, there was a notable reduction in phosphorylated ERK (extracellular signal-regulated kinase), phosphorylated GSK-3β, and phosphorylated S6K (a downstream target of mTORC1) in all cell lines. However, prolonged trastuzumab inhibition led to an increase in the phosphorylation of ERK, GSK-3β, and S6K (Fig. 3g). In summary, these results suggest that trastuzumab induces chromatin remodeling and target gene transcription in HER2-positive cells characterized by enhancer inhibition followed by reactivation, and that YAP/TEAD binding and mTOR signaling reactivation occur in chromatin regulatory elements, indicating that YAP and mTOR signaling are promising joint targets for overcoming trastuzumab-adaptive resistance.

### YAP inhibition specifically enhances the cancer cell killing effects of trastuzumab by decreasing the activity of the PI3K/mTOR axis

**Fig. 4:**
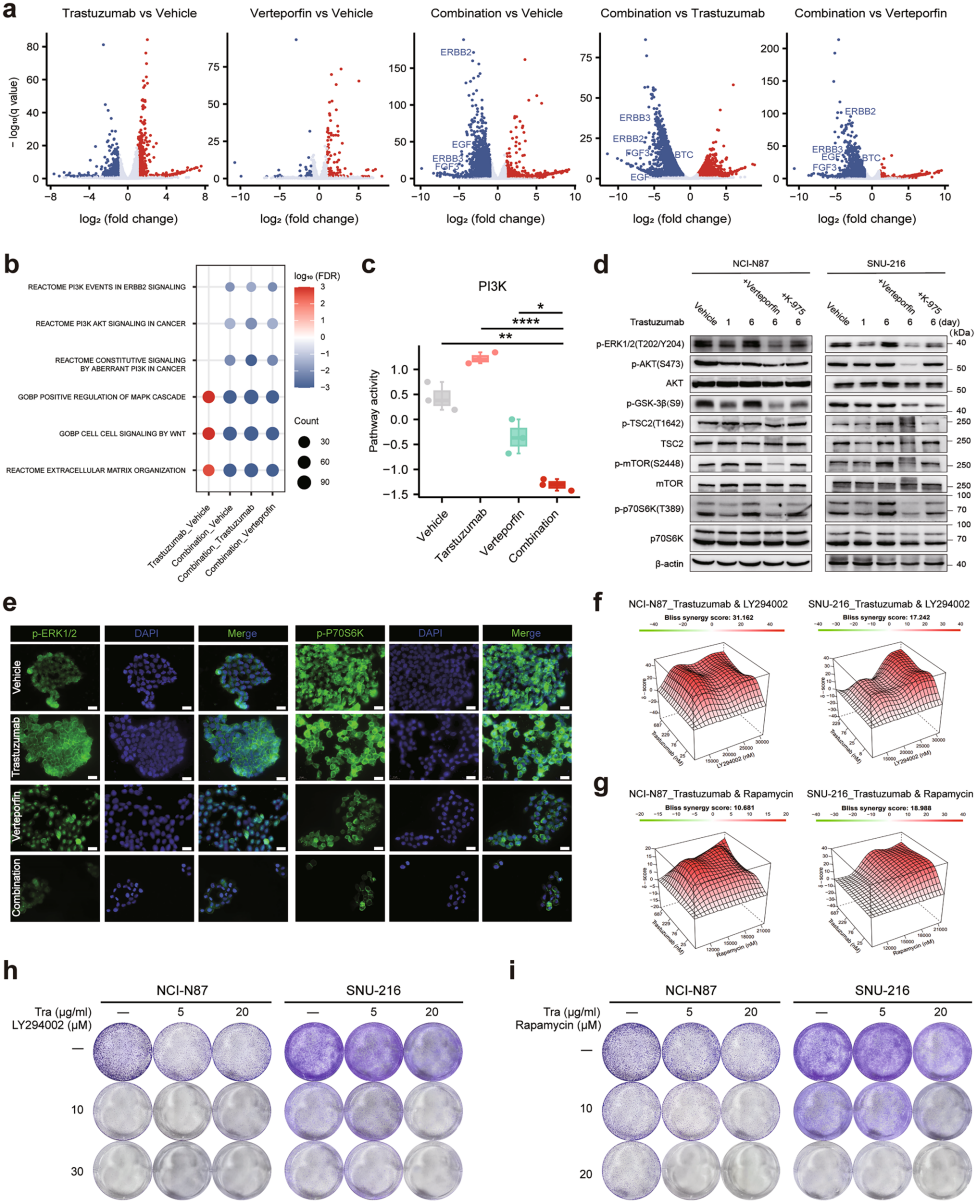
YAP regulates the AKT/mTOR and ERK/mTOR signaling pathways, and the combination of trastuzumab with YAP or mTOR inhibitor exhibits significant synergistic effect. **a** Volcano plot of − log10 (q value) versus log2 (fold change) of differentially expressed genes (DEGs) after 24 h of trastuzumab treatment. Genes whose expression increased are colored in red, and genes whose expression decreased are colored in blue. Differential gene expression was normalized by DESeq, and adjusted P values were analyzed by Benjamini and Hochberg's approach. **b** Dot plot of representative enriched pathways in different comparisons of NCI-N87 cells. Pathways enriched in upregulated genes are colored in red and downregulated genes are in blue. **c** Box plots showing the activity levels of PI3K signaling in the four cohorts of NCI-N87 cells. **d** Western blot analysis of ERK1/2, AKT, GSK-3β, TSC2, mTOR, and p70S6K phosphoprotein levels and total AKT, TSC2, mTOR, and p70S6K total protein levels in NCI-N87 (left) and SNU-216 (right) cells treated for 6 d with 30 μg/ml trastuzumab alone or in combination with 1 μM verteporfin or 10 μM K-975 for 24 h. **e** The localization of p-ERK1/2 and p-p70S6K in NCI-N87 cells treated with trastuzumab for 24 h, with or without verteporfin treatment, was analyzed by immunofluorescence staining (scale bar = 50 µm). p-ERK1/2 and p-p70S6K were stained with Alexa Fluor 488-conjugated IgG (green), and the nuclei were stained with DAPI (blue), n = 3 independent experiments. **f** Synergistic model and scores of trastuzumab and rapamycin in NCI-N87 (left) and SNU-216 (right) cells. **g** Synergistic model and scores of trastuzumab and LY294002 in NCI-N87 (left) and SNU-216 (right) cells. **h** NCI-N87 and SNU-216 cells were treated with trastuzumab (5 or 20 μg/ml), LY294002 (10 or 30 μM), or a combination of trastuzumab and LY294002 and subjected to colony formation assays. **i** NCI-N87 and SNU-216 cells were treated with trastuzumab (5 or 20 μg/ml), rapamycin (10 or 20 μM), or a combination of trastuzumab and rapamycin for 2 weeks, after which colony crystal violet staining was performed

To investigate the mechanism underlying the synergistic lethality of the combination of YAP inhibitors and trastuzumab, we performed the transcriptomic profiling of NCI-N87 and SNU-216 cells subjected to four different treatment regimens (vehicle, trastuzumab, verteporfin, or the combination). Significant changes were observed in both the upregulated and downregulated genes in the combination group compared to either the vehicle or the monotherapy groups (Fig. 4a, Fig. S4a). Consistent with our findings above, pathway and GO-BP enrichment analyses revealed that the PI3K/AKT pathway and MAPK pathway were downregulated compared with those in the vehicle or monotherapy groups (Fig. 4b, c, Fig. S4b, c). Western blot analysis demonstrated that the combination of verteporfin or K-975 attenuated the reactivation of p-AKT, p-mTOR, and p-P70S6K in NCI-N87 and SNU-216 cells induced by prolonged trastuzumab stimulation (Fig. 4d). Since activation of mTOR signaling can also occur via ERK1/2, we assessed the pharmacological cross talk between ERK, trastuzumab, and YAP in the NCI-N87 and SNU-216 cells. Both YAP inhibitors, verteporfin and K-975, effectively blocked trastuzumab-induced feedback phosphorylation of S6K and inhibited ERK phosphorylation (Fig. 4d). The immunofluorescence results also confirmed that the combination therapy decreased the expression levels of p-ERK and p-P70S6K in NCI-N87 cells (Fig. 4e).

To evaluate the sensitivity of HER2-positive cells to a PI3K inhibitor (LY294002) and an mTOR inhibitor (rapamycin), dose–response experiments were performed on NCI-N87 and SNU-216 cells. In NCI-N87 cells, the IC50 values for LY294002 and rapamycin were 17.39 μM and 16.45 μM, respectively. For SNU-216 cells, the IC50 values were 85.51 μM for LY294002 and 17.05 μM for rapamycin (Fig. S4d–f). Furthermore, we assessed the therapeutic effectiveness of combining LY294002 or rapamycin with trastuzumab to inhibit the proliferation of HER2-positive cells. Bliss synergy analysis revealed cooperative interactions between trastuzumab and LY294002 (or rapamycin) in both NCI-N87 and SNU-216 cells (Fig. 4f, g, Fig. S4g, h). Additionally, the colony formation assay demonstrated that the combination of trastuzumab and LY294002 or rapamycin markedly inhibited the growth of NCI-N87 and SNU-216 cells (Fig. 4h, i). In conclusion, it has been demonstrated that YAP, PI3K, or mTOR inhibitors can increase the therapeutic efficacy of trastuzumab in HER2-positive GC.

### Molecular mechanisms of YAP inhibition-mediated suppression in HER2-positive GC cells

**Fig. 5:**
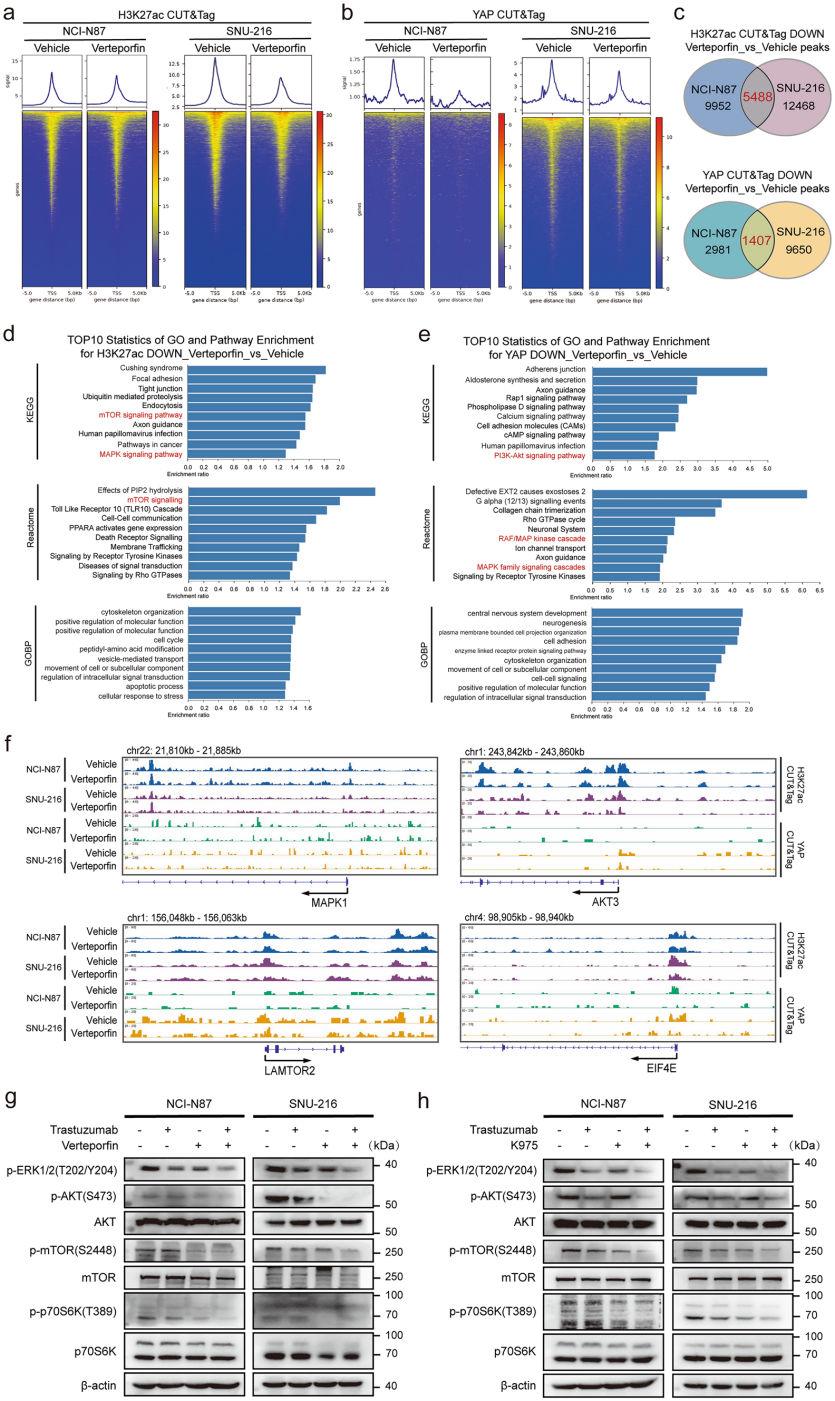
Inhibition of YAP derepresses the AKT/mTOR and ERK/mTOR signaling pathways in HER2-positive GC cells. **a**, **b** A TSS heatmap showing that the H3K27ac and YAP signals were significantly lower after verteprofin treatment for 24 h than after vehicle treatment. **c** Venn diagram showed the number of peaks coregulated by H3K27ac (topper) and YAP (lower) after the addition of Verteporfin in NCI-N87 and SUN-216 cells, respectively, compared with the Vehicle group. **d** The common genes with decreased H3K27ac levels in NCI-N87 and SNU-216 cells treated with vehicle for 24 h compared with vehicle were subjected to KEGG, Reactome, and GO enrichment analyses. **e** The common downregulated YAP peaks in NCI-N87 and SNU-216 cells with Vehicle for 24 h compared with Vehicle were analyzed for KEGG, Reactome, and GO enrichment. **f** Genome browser tracks of H3K27ac (upper) and YAP (lower) CUT& Tag signals at representative target gene loci. **g**, **h** Western blot analysis of ERK1/2, AKT, mTOR, and p70S6K phosphoprotein levels and total AKT, mTOR, and p70S6K protein levels in NCI-N87 and SNU-216 cells treated with 30 μg/ml trastuzumab alone or in combination with 1 μM verteporfin (left) or 10 μM K-975 (right) for 24 h

To determine whether the decrease in PI3K/mTOR signaling upon YAP inhibition is driven by chromatin alterations, we next investigated the landscape of active enhances by H3K27ac and YAP CUT&Tag in NCI-N87 and SNU-216 cells over 24 h of verteporfin treatment. We observed an overall decrease in H3K27ac and YAP CUT&Tag signals after verteporfin treatment (Fig. 5a, b), and KEGG, Reactome, and GO-BP enrichment analyses revealed the downregulation of the PI3K–AKT, MAPK, and mTOR signaling pathways in two cells (Fig. 5c–e). CUT&Tag tracks H3K27ac signals and YAP binding sites with MAPK1, AKT3, LAMTOR2, and EIF4E in NCI-N87 and SNU-216 cells that lost YAP binding activity, both in the presence of vehicle or verteporfin (Fig. 5f). In addition, the H3K27ac signals of MAPK1, AKT3, PIK3CA, and RPS6KB1 were reduced after combination therapy (Fig. S5). Consistent with the CUT&Tag findings, western blot analysis also indicated that the YAP inhibitor combined with trastuzumab therapy contributed to GC progression via the AKT/mTOR and ERK/mTOR pathways (Fig. 5g, h). Overall, we found that verteporfin inhibition represses the chromatin recruitment of H3K27ac and YAP, leading to the remodeling of regulatory regions of mTOR signaling pathways and causing an epigenetic switch to YAP transcriptional dependency.

### YAP inhibitor combined with trastuzumab improves anti-tumor efficacy in the CDX model

**Fig. 6:**
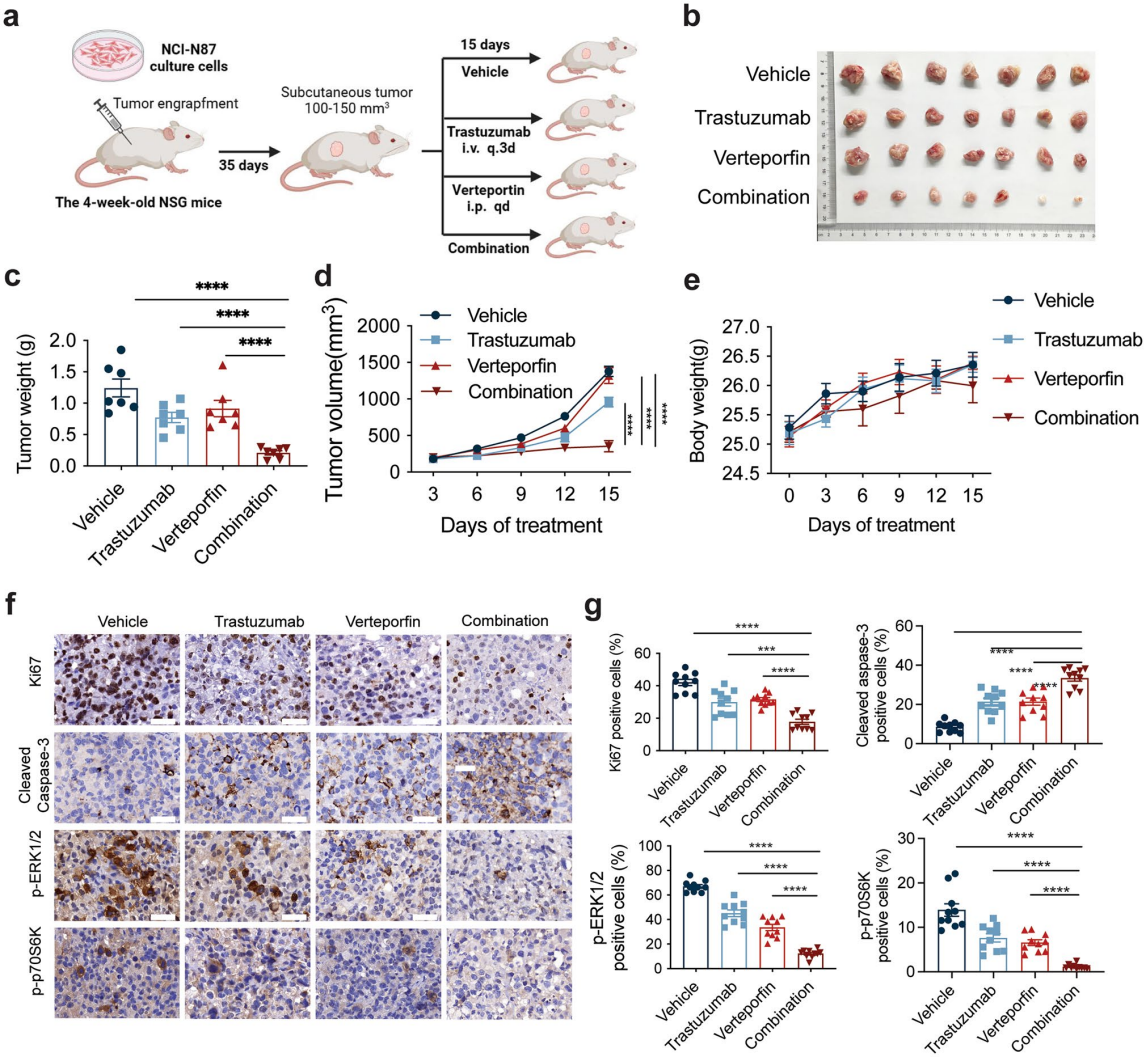
Verteporfin synergized with trastuzumab in vivo by inducing cell apoptosis and the mTOR signaling pathway. **a** Schematic of the method used to evaluate the anti-tumor efficacy of trastuzumab and verteporfin in vivo. NSG tumor-bearing mice received vehicle (solvent control), trastuzumab (15 mg/kg every three days, intravenously), verteporfin (2 mg/kg/day, intraperitoneal injection), or a combination of both for 15 days. The schematic was created with BioRender.com with permission. **b** Representative images of xenograft tumors obtained from the indicated groups at the endpoint of the experiments. **c** Histogram showing the mean ± SEM of the tumor weights from the indicated groups at the endpoint of the experiments (*n* = 7 mice/group). **d** Line chart showing the tumor volumes in the indicated groups, which were measured and are presented as the mean ± SEMs (*n* = 7 mice/group). **e** Line chart showing the weight of mouse in the indicated groups (*n* = 7 mice/group). **f** Representative images at the end of the study of tumors showing the immunohistological evaluation of cellular proliferation by Ki-67 staining, cellular apoptosis by cleaved caspase-3, and markers of p-ERK1/2, and p-p70S6K, each of which were labeled in the treatment cohort. Scale bar = 50 μm. **g** Quantification of the percentage of positive cells was performed using the ImageJ plugin (IHC Profiler) of ImageJ software (n = 10). The data are presented as the mean ± SEM of ten replicates. ***P < 0.001, ****P < 0.001 versus the combination group

To evaluate the in vivo efficacy of trastuzumab combined with verteporfin, we constructed CDX models using the NCI-N87 cell line (Fig. 6a). Compared with either monotherapy, the combination of trastuzumab and verteporfin significantly restricted the tumor burden and progression in the CDX model (Fig. 6b, c), resulting in a sharp reduction in tumor volume (Fig. 6d). Notably, the treatment was well tolerated in the CDX model, with no adverse effects on mouse weight observed during administration (Fig. 6e). Additionally, immunohistochemistry (IHC) analysis of tumors isolated from mice revealed significant inhibition of Ki67 expression in the combination treatment cohort compared with that in the single agent group (Fig. 6f). These collective results demonstrate the enhanced in vivo anti-tumor efficacy of YAP inhibitors combined with trastuzumab.

We performed immunohistochemical analyses on tumor tissues from four treated groups to assess whether YAP inhibitor combined with trastuzumab therapy, which acts through the AKT/mTOR and ERK/mTOR pathways can be replicated in vivo. The results indicated a significant reduction in cell proliferation and a notable decrease in AKT/mTOR and ERK/mTOR signaling in tumors subjected to combination therapy (Fig. 6f, g). Overall, these preclinical studies validated the potential of combining trastuzumab with YAP or mTOR inhibitors.

## Discussion

Clinically, anti-HER2-targeted therapy is determined based on HER2 expression detected by fluorescence in situ hybridization (FISH) and immunohistochemistry (IHC) [32]. Despite trastuzumab plus chemotherapy being the standard treatment for patients with advanced metastatic HER2-positive GC, not all of these treatments are beneficial with an overall response rate of only 47% (5% complete response plus 42% partial response) [13]. In addition, trastuzumab resistance poses a significant challenge for the clinical treatment of patients with HER2-positive metastatic GC. Several approaches, including circulating tumor DNA detection and gene expression profiling, have been used to study trastuzumab resistance [33]. In HER2-positive GC resistant to trastuzumab, glycolysis fluctuates in tandem with the circadian rhythm regulated by the BMAL1–CLOCK–PER1–HK2 axis. Introducing compounds that regulate the circadian clock into treatment strategies is proposed as a strategy to overcome resistance to trastuzumab [34]. In addition, researchers have explored the development of CD40 × HER2 bispecific antibodies (bsAbs), which possess CD40-targeted macrophage co-stimulatory activity and HER2-mediated anti-tumor activity, with limited immune-related adverse events (irAEs), offering a partial solution to trastuzumab resistance in GC [35]. However, the underlying mechanism of trastuzumab resistance in HER2-positive GC remains unclear.

**Fig. 7:**
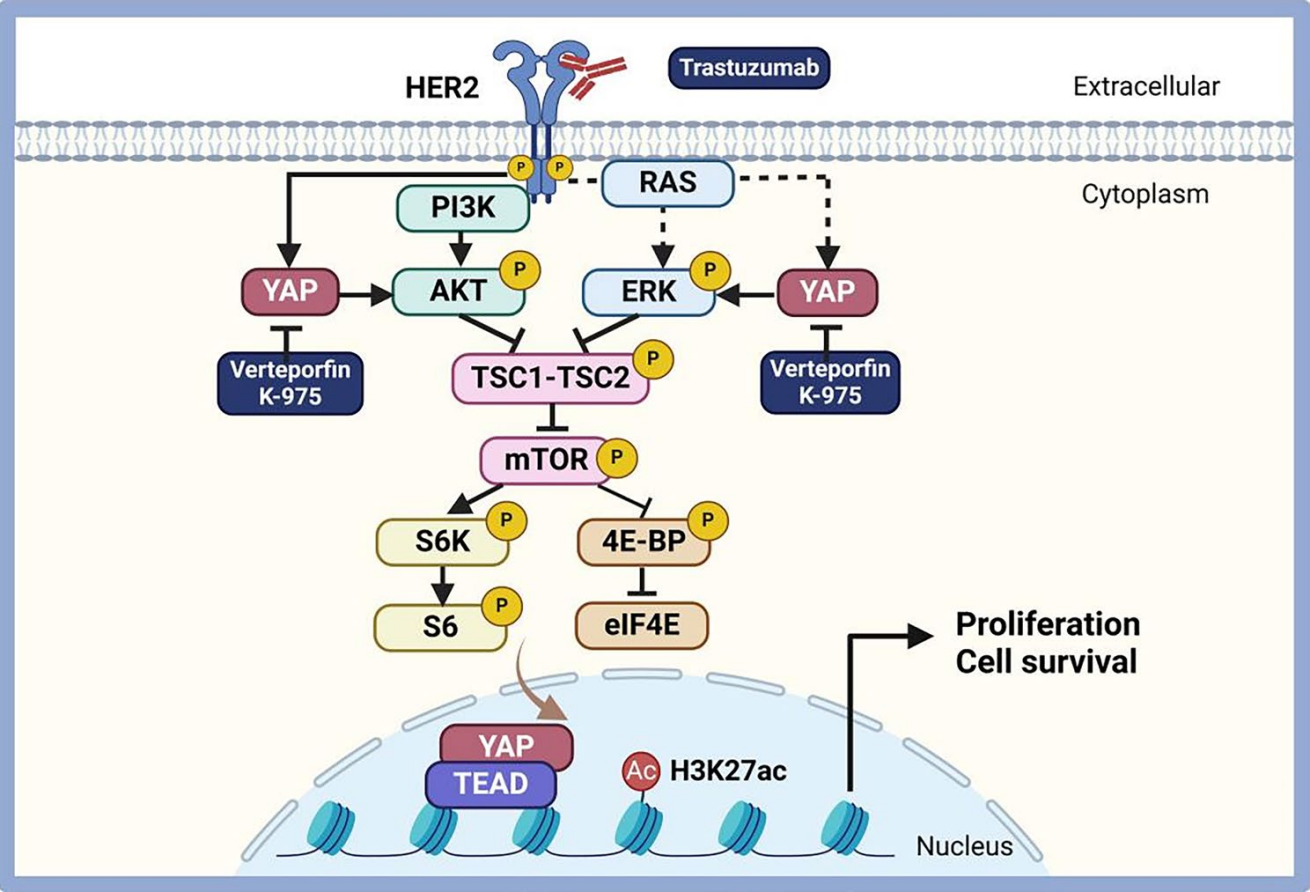
Schematic illustration of the mechanism by which YAP inhibition attenuates adaptive resistance to trastuzumab in HER2-positive GC cells. The schematic was created with BioRender.com with permission

In this study, for the first time, we elucidated the role of the HER2–YAP–mTOR axis in trastuzumab resistance in HER2-positive GC (Fig. 7). Using the HER2-positive GC cell lines NCI-N87 and SNU-216, along with CDX mouse models, we demonstrated that the combination of a YAP inhibitor and trastuzumab leads to growth inhibition and cycle arrest in HER2-positive GC cells, enhancing anti-tumor efficacy in mice. In addition, reactivation of the AKT/mTOR and Erk/mTOR signaling pathways is required for adaptive resistance to trastuzumab. In summary, these results provide the basis for preclinical studies of the use of YAP inhibitors and trastuzumab for the treatment of HER2-positive GC patients.

Dysregulation of the Hippo signaling pathway has been detected in various solid tumors, including GC, with implications for tumor proliferation, metastasis, and drug resistance [36, 37]. Notably, YAP expression and nuclear localization are significantly greater in primary and metastatic GC than in areas with normal gastric epithelial hyperplasia [38]. Nevertheless, the correlation between YAP and trastuzumab resistance in HER2-positive GC cells has not been fully elucidated. Cao et al. elucidated potential mechanisms of trastuzumab resistance in HER2-positive breast cancer and demonstrated the utility of YAP and p-AKT in predicting the response to neoadjuvant chemotherapy comined with trastuzumab in breast cancer patients [25]. A recent study elucidated that another member of the ERBB family, HER4, also influences the survival of HER2-positive cancer cells after they develop resistance to trastuzumab, and network analysis utilizing STRING revealed that YAP1 is predicted to be a crucial downstream molecule that induces epithelial–mesenchymal transition (EMT) [26]. Therefore, YAP has emerged as a promising drug target for the treatment of GC. Verteporfin, a representative inhibitor that blocks the YAP–TEAD complex, has been shown to inhibit the proliferation of gastric cancer stem cells (CSCs) and suppress tumor growth in vivo in xenotransplantation models of GC patients [39]. In NCI-N87 cells, verteporfin effectively inhibited cell growth and proliferation, increasing apoptosis and causing most cells to undergo cell cycle arrest in the G0/G1 phase and significantly reducing the number of cells in the S phase. When verteporfin was combined with trastuzumab, the dual therapy exhibited a synergistic effect on apoptosis and proliferation. The CDX model offers good controllability and consistency, enabling high-throughput drug screening and investigation of drug resistance mechanisms and providing a relatively simplified tumor model. Although the CDX model may not accurately reflect the complexity and heterogeneity of patient tumors as well as the PDX model, it remains a valuable experimental tool, providing insights for further research. In vivo studies further validated the superior anti-tumor efficacy of the combination compared to that of monotherapy, with no significant adverse reactions observed in treated mice.

In contrast to previous studies on trastuzumab resistance, our study revealed that, under prolonged trastuzumab exposure, YAP-dependent activation mediates the reactivation of the mTOR signaling pathway. These findings align with existing research on the role of mTOR and YAP [40, 41]. Therefore, the interplay between mTOR and YAP transcriptional regulation may thus play an important role in tumor progression and the drug response in HER2-positive GC.

### Supplementary Information

Below is the link to the electronic supplementary material.

Supplementary file 1 (DOCX 34 kb)

Supplementary file 2 (DOCX 1366 kb)

Supplementary file 3 (TXT 2565 kb)

Supplementary file 4 (TXT 2563 kb)

Supplementary file 5 (PDF 2516 kb)

## Abbreviations

bsAbs: Bispecific antibodies
CDX: Cell line-derived xenografts
CSCs: Gastric cancer stem cells
CUT&Tag: Cleavage under targets and tagmentation
DAPI: 4',6-Diaminyl-2-phenylindoles
GC: Gastric cancer
H3: Human CRISPR knockout library
HER2: Human epidermal growth factor receptor 2
IHC: Immunohistochemical
irAEs: Immune-related adverse events
LAMTOR1: MTOR activator 1
LAMTOR5: MTOR activator 5
ORR: Objective response rate
OS: Overall survival
TEAD: Transcriptional enhanced associate domain
ToGA: Trastuzumab for gastric cancer
YAP: YES-associated protein
