# Supplementary material for: YAP inhibition overcomes adaptive resistance in HER2-positive gastric cancer treated with trastuzumab via the AKT/mTOR and ERK/mTOR axis
Source: Gastric Cancer. 2024 May 23;27(4):785–801. doi: 10.1007/s10120-024-01508-3 (PMC11193831; doi:10.1007/s10120-024-01508-3)
Supplement: Supplementary file 1 — (DOCX 34 kb) [file 10120_2024_1508_MOESM1_ESM.docx]

**Supplementary Materials and Methods**

1. **Cell lines, drugs, antibodies, and assay reagents**

The following cell lines were purchased from ATCC: NCI-N87 and AGS. SGC-7901, MGC-803, and MKN-45 cells were purchased from CSIC. HGC-27 cells were obtained from ECACC. SNU-216 cells and GES-1 cells were purchased from MeisenCTCC. Seven human GC cell lines and normal GES-1 gastric mucosa epithelium cell lines were cultured at 37 °C with 5% CO2. All culture medium reagents were obtained from Gibco, USA. Detailed cell source information is included in Table S1. Trastuzumab (HY-P9907), verteporfin (HY-B0146), and K-975 (HY-138565) were obtained from MedChemExpress. For *in vitro* experiments, except for trastuzumab, which was dissolved in 10 mg/ml PBS, the other compounds were dissolved and preserved in DMSO (10 mM). Next, the compounds were diluted in the medium and added to each well to the specified concentration. The final concentration of DMSO in the medium was not greater than 4‰. Cell Counting Kit-8 regents (C0005) was purchased from TargetMol. Crystal violet was obtained from Sigma. Antibodies against HER2/ErbB2 (#2165S), YAP (#14074S), phospho-Akt (Ser473) (#4060T), phospho-AKT (Thr308) (#13038T), AKT (#4691T), phospho-p44/42 MAPK (Erk1/2) (Thr202/Tyr204) (#4370T), phospho-GSK-3β (Ser9) (#5558T), phospho-Tuberin/TSC2 (Thr1462) (#3617S), TSC2 (#4308P), phospho-mTOR (Ser2448) (#5536T), mTOR (#2983S), phospho-p70 S6 kinase (Thr389) (#9234S), and p70 S6 kinase (#2708S) were purchased from Cell Signaling Technology. Antibodies against phospho-p44/42 MAPK (Erk1/2) (Thr202/Tyr204) and phospho-p70 S6 Kinase (Thr389/Thr412) (#AF3228) were obtained from Affinity Biosciences. Anti-β-actin (A0101) antibody was purchased from Lablead. The Ki-67 rabbit mAb (#15,580) was purchased from Abcam. HRP-conjugated goat Anti-rabbit IgG (H + L) secondary antibody (S0101) and goat anti-mouse IgG (H + L) secondary antibody (S0100) were purchased from Lablead. Goat anti-rabbit IgG H&L (Alexa Fluor® 488) (ab150077) antibody was obtained from Abcam.

1. **Genome-wide CRISPR knockout screening**

The human CRISPR knockout library (H3) was a gift from Xiaole Shirley Liu and Myles Brown. One hundred million NCI-N87 cells were transduced with the H3 library at a low infective multiplicity (MOI) (≈0.3) so that most cells received only one lentiviral-directed RNA. Fourty-eight hours after infection, the cells were selected with puromycin. After approximately 3-5 days, an initial pool of 30 million NCI-N87 cells was harvested for genomic DNA extraction using the
QIAamp DNA Blood Midi Kit (Qiagen, #51183) according to the manufacturer’s protocol. The other cells (treated with trastuzumab or vehicle) were screened, passaged, collected after 14 days, and harvested for genomic DNA. The library was prepared by three rounds of polymerase chain reaction, and the sample was purified with the TIANgel Purification Kit (Tiangen, #DP219) and sequenced on the Illumina NovaSeq 6000 platform. The experiment was repeated once. Data from genome-wide CRISPR KO screenings were analyzed using the MAGeCKFlute algorithm, as previously published[25]. MAGeCKFlute combines the MAGeCK and MAGeCK-VISPR algorithms, followed by downstream functional analysis using FluteMLE.

**3. Dose-dependent assay**

A 100 μL cell suspension (2 × 103 cells per well) was prepared in a 96-well plate and pre-cultured in an incubator for 24 h (37 ℃, 5% CO2). Then, 10 μL of different concentrations of drugs were added to the culture plate and treated for 3 days. Ten microlitres of CCK-8 reagent was added to each well, and the plates were incubated for 2 h. The absorbance at 450 nm was measured with an enzyme marker. Using GraphPad Prism 9, we imported the drug concentration and corresponding cell viability data. Drug concentration was used as the independent variable, and cell viability was used as the dependent variable. The independent variable was logarithmically transformed to the base 10 of the drug concentration. We then applied the four-parameter logistic model to fit the drug concentration-effect curve. After performing curve fitting in Prism, we obtained fitting parameters, including the IC50 value.

**4. Drug synergy analysis**

The drug synergy trial was conducted in a checkerboard format, in which we evaluated the cell viability of five doses of trastuzumab versus five doses of verteporfin (36 different dose combinations). After 72 hours of exposure, CCK-8 regents was added, and the absorbance at 450 nm was measured using an enzyme marker. Relative cell numbers (expressed as survival rate %) were calculated. Drug synergy scores were based on the Bliss model and determined using the R software package SynergyFinder.

**5. Colony formation assay**

NCI-N87 and SNU-216 cells (2×10^3^) were inoculated in six-well plates and treated with trastuzumab (0, 1 μg/ml, 10 μg/ml, or 30 μg/ml) in combination with verteporfin (0, 0.03 μM, 0.06 μM, or 0.09 μM). The medium and drugs were replaced every 3 days. After 14 days of drug treatment, the cells were washed once with PBS, fixed with 4% paraformaldehyde at room temperature for 30 min, stained with 0.1% crystal violet for 15 min, washed with PBS several times, and dried after which images were taken.

**6. Cell cycle assay**

After 48 hours of treatment, the NCI-N87 cells were collected and gently re-suspended in PBS. According to the instructions in the [Cell Cycle and Apoptosis Kit](http://www.baidu.com/link?url=FGCJA_fwgwQ2jFs0FYX7BH_5lsIQ85piQf3c0END0kCxqhiqYEBT4vIWQOK3naQLVK2ztdnZpn_eGdE8hYLAaK) (Beyotime Biotechnology, code: C1052), 1 ml of pre-cooled 70% ethanol was added, and the mixture was gently blown and mixed at 4 ℃ overnight. After centrifuge at 1000 × g for 3 minutes, 0.5 ml of propyl iodide staining solution was added to the precipitated cells to re-suspend the cell precipitate, and the mixture was incubated at 37 ℃ for 30 minutes in the dark. Flow detection and analysis were performed with ModfitLT 5.

**7. L**[**entiviral vector transfection**](javascript:;)

The YAP lentivirus plasmid was purchased from Mailgene Biosciences Co., Ltd. with the following sequence: YAP shRNA-1, 5’-CCGGCAGGTGATACTATCAACCAAACTCGAGTTTGGTTGATAGTATCACCTGTTTTTG-3’; YAP shRNA-2, 5’-CCGGGACCAATAGCTCAGATCCTTTCTCGAGAAAGGATCTGAGCTATTGGTCTTTTTG-3’; and YAP shRNA-3, 5’-CCGGGCCACCAAGCTAGATAAAGAACTCGAGTTCTTTATCTAGCTTGGTGGCTTTTTG-3’. According to the manufacturer's instructions, lentivirus was generated in 293T packaging cells. NCI-N87 and SNU-216 cells were transfected with the lentivirus and treated with 5 μg/mL polybrene. Cells were selected with 1 μg/mL puromycin 24 h after lentivirus infection and assessed in vitro 5 days later.

**8. Western blotting**

Total proteins from cells and tissues were collected by adding 1% protease (Shanghai Epizyme Biomedical Technology Co., Ltd, GRF101) and 1% phosphatase inhibitor (GRF102) to RIPA lysis buffer (Applygen Technologies Inc., C1053-100). YAP cytoplasmic and nucleoprotein were extracted using a membrane/cytoplasmic/nucleoprotein extraction kit (Beijing Solarbio Science & Technology Co., Ltd., EX1400). Total protein concentrations were measured using a BCA protein assay kit (Invitrogen, 23227). The samples were separated on a 10% SDS-PAGE gel, and bands from the gel were subsequently transferred to a PVDF membrane (Millipore, IPVH00010). The membrane was incubated in 5% skim milk containing 0.05% Tween 20 (TBST) at room temperature for 1 h, incubated with primary antibody (1:1000 dilution) overnight at 4 °C, washed three times in TBST, and incubated with horseradish peroxidase-conjugated secondary antibody (1:5000 dilution). Finally, the proteins were visualized by enhanced chemiluminescence (ECL) using a 1:1 Pierce ECL Western blotting substrate (Millipore, WBKLS0500). ImageJ software was used to analyze the signal quantitatively.

**9. Immunofluorescence analysis**

NCI-N87 cells were seeded at a density of 2×10^5^ cells per well on a six-well plate for immunofluorescence. After different treatments, the cells were fixed with 4% paraformaldehyde for 10 min, permeated with 0.3% Triton X-100 at room temperature for 5 min, and then sealed in 5% BSA/PBS at room temperature for 30 min. Overnight incubation was performed with the corresponding antibodies against p-P70S6K (1:100), and p-ERK1/2 (1:100). The cells were then washed and incubated at room temperature with goat anti-rabbit IgG H&L (Alexa Fluor® 488) (1:200) for 1 hour. The nuclei were stained with 4',6-diamidino-2-phenylindole (DAPI, Sigma-Aldrich). A Pannoramic MIDI (3D HISTECH) instrument was used to collect images.

**10. RNA-seq data processing and analysis**

NCI-N87 cells were inoculated in a six-well plate and treated for 1 d with vehicle, 10 μg/ml trastuzumab, 1 μM verteporfin, or a combination of two drugs. RNA was extracted from NCI-N87 cells by TRIzol method, and all experiments were repeated three times. The RNA-seq library was generated using the NEBNext® Ultra RNA Library Prep Kit for Illumina (NEB, #E7530). All the samples were sequenced using the Illumina NovaSeq 6000 platform and PE150 sequencing strategy. After CASAVA base recognition, the sequence data were converted into fastq format. Use STAR + SAMtools + DESeq2 was used for read comparison, quality control, and data analysis. The sequencing data were aligned to the GRCh38 human genome using STAR2 v2.7.3a, and then the transcripts were quantified using RSEM (1.3.3)[26]. DEseq2 (1.20.0) was used for differential expression analysis. GO and KEGG pathway enrichment analyses of the differentially expressed genes were performed using ClusterProfiler (3.8.1).

**11. ATAC-seq**

Fifty thousand NCI-N87 cells were taken from each group, washed once with PBS, and resuspended in cold lysis buffer solution (10 mM Tris-HCl, pH 7.4, 10 mM NaCl, 3 mM MgCl2, 0.1% NP-40)[27]. It was disrupted by the Tn5 transposition enzyme (Vazyme, #TD501). Purified DNA was obtained by purification of the fragment products, which were amplified and barcoded with the TruePrepTM Index Kit V2 for Illumina® (Vazyme, #TD202). VAHTSTM DNA Clean Beads were used for length sorting of the amplified products, and the library quality was analyzed using a BioAnalyzer 2100. Sequencing was performed using the Illumina NovaSeq 6000 platform.

**12. CUT&Tag-Seq**

NCI-N87 cells were collected, counted, and centrifuged at 600×g for 5 min. Then, 1 × 105 cells were gently washed once in 500 μL of washing buffer. The ConA beads were activated by magnetic beads. Ten microliters of activated magnetic beads were added to each sample and incubated at room temperature for 10 min. According to the kit instructions, the cells were sequentially incubated with ConA beads, primary antibodies (anti-H3K27AC antibody, anti-Yap antibody), secondary antibodies, and hyperactive pA/G-Tnp transposons, and then fragmented. Fragments of DNA were extracted from the samples, and amplified by PCR, and the products were subsequentaly purified. A CUT&Tag library was constructed using a Hyperactive In-Situ ChIP Library Prep Kit for Illumina (Vazyme, #TD901), and sequenced on an Illumina NovaSeq 6000 platform.

**13. Xenograft studies**

All mouse experiments were approved by the Animal Health and Use Committee of Peking University First Hospital (No. 202158). Four-week-old male NSG mice were purchased from Beijing Vital River Laboratory Animal Technology Co., Ltd., and were bred and housed under specific pathogen-free conditions according to the guidelines established in the National Institutes of Health Guidelines for the Care and Use of Laboratory Animals[28]. A 0.2 mL volume of the NCI-N87 cell suspension was injected subcutaneously into the shoulder of each group (5 × 10^6^ cells/mouse). The tumor size was monitored with a digital caliper every three days. the tumor volumes were calculated by the formula: Volume = 1/2 × Length × Width^2^. When the tumor volume reached 100 mm^3^, the mice were randomly divided into 4 groups (n=7 per group): (1) vehicle (PBS); (2) trastuzumab monotherapy (15 mg/kg every three days, intravenously); (3) verteporfin monotherapy (2 mg/kg/day, intraperitoneal injection); and (4) trastuzumab combined with verteporfin. The treatment study lasted for 2 weeks. The animals were euthanized on Day 15, after which the tumors were removed, measured, weighed, and photographed.

**14. Histopathology, and immunohistochemistry of paraffin samples**

Mouse gastric tissue was fixed overnight in 4% formalin. The samples were embedded in paraffin wax and sliced into 5 μm thick sections. After blocking with 30% goat serum for 25 min at 4 °C, antigen repair was performed by thermal intercalation in citrate buffer (pH 6.0). Cleaved caspase-3, p-P70S6K, and p-ERK1/2 were stained overnight at 4 ℃. A goat anti-rabbit biotin secondary antibody was used to detect the primary antibody and the DAB substrate reagent was added for direct color visualization. To detect effect of treatment on cell proliferation, dewaxed and hydrated sections were stained with an anti-Ki67 antibody (2 μg/mL).

**15. Public datasets**

Immunohistochemical staining of HER2 was obtained from the Human Protein Atlas. The differential expression of HER2 in STAD tumors and normal tissues was analyzed with GEPIA 2. Pan-cancer genome-dependency data were obtained from the DepMap portal. Survival analysis data were collected from the Kaplan-Meier Plotter Database, and drug target screening was performed through Therapeutic Target Database and PharmSnap Database.

**16. Statistical analysis**

Data are expressed as the mean ± standard deviation. Each experiment was repeated at least three times. Comparisons were made with Student's t-test. Statistical significance was evaluated by GraphPad Prism 9 software (La Jolla, CA, USA). A value of *P* < 0.05 was considered to indicate statistical significant.
