# Supplementary material for: YAP inhibition overcomes adaptive resistance in HER2-positive gastric cancer treated with trastuzumab via the AKT/mTOR and ERK/mTOR axis
Source: Gastric Cancer. 2024 May 23;27(4):785–801. doi: 10.1007/s10120-024-01508-3 (PMC11193831; doi:10.1007/s10120-024-01508-3)
Supplement: Supplementary file 2 — (DOCX 1366 kb) [file 10120_2024_1508_MOESM2_ESM.docx]

Supplementary Materials for

**YAP Inhibition Overcomes Adaptive Resistance in HER2-Positive Gastric Cancer Treated with Trastuzumab via AKT/mTOR and ERK/mTOR Axis**

Jiao Qiao *et al.*

*Corresponding author. Email: Liyan Cui, [cliyan@163.com](mailto:cliyan@163.com)

**This file includes:**

Fig. S1 to S5

Table S1


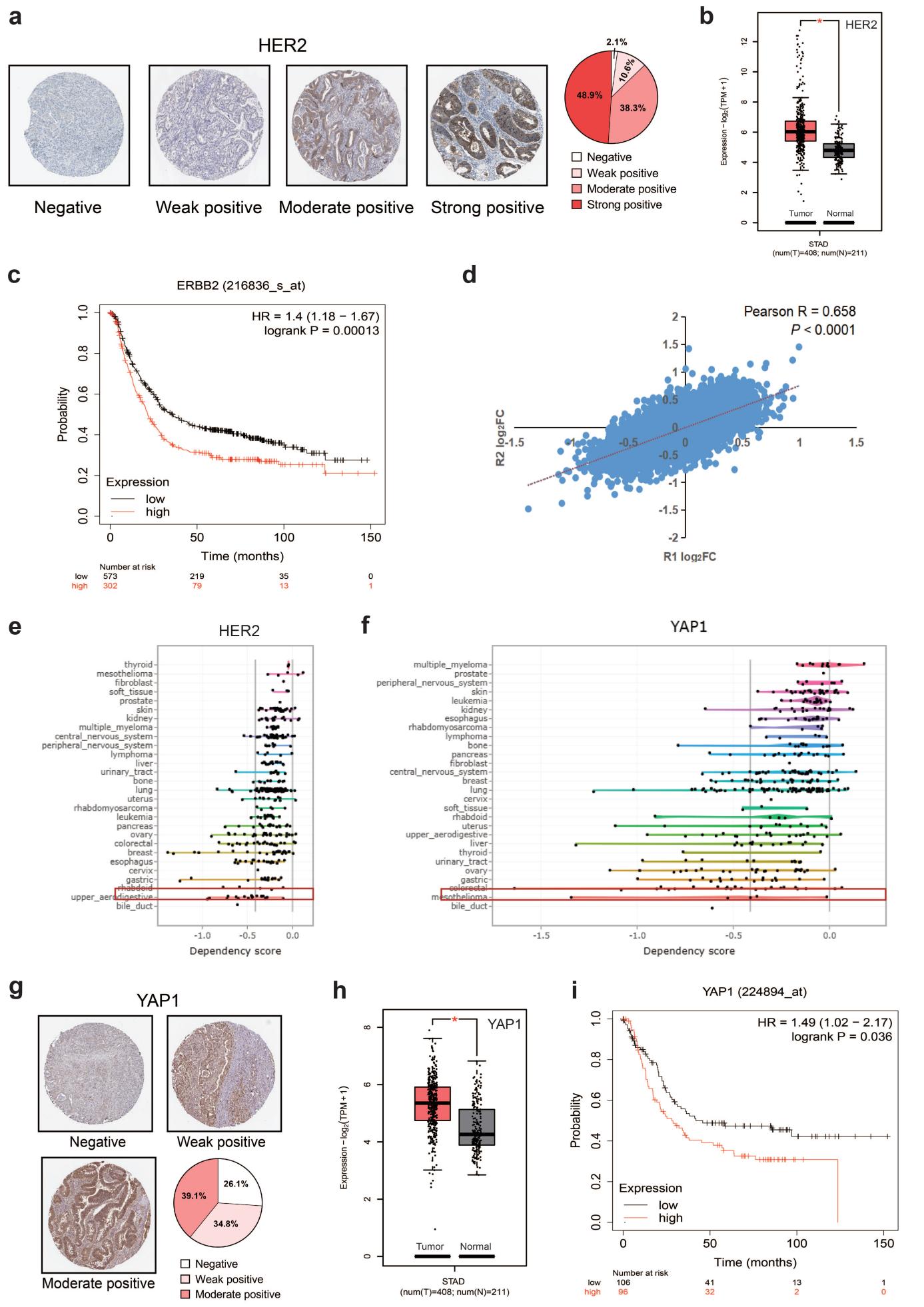


**Fig. S1. Analysis of the sensitivity of HER2-positive GC cells to trastuzumab and pharmacological inhibition of YAP. a** Immunohistochemical staining of HER2 in GC patients from The Human Protein Atlas. **b** Transcriptional expression level of HER2 between tumor and normal tissues in the TCGA-STAD cohort. Tumor number = 408, normal number = 211. * *P* <0.05 vs. normal. **c** Kaplan-Meier curves from the Kaplan-Meier Plotter Database of time for probability showing the difference between GC patients with low and high HER2 levels. n = 875, log-rank test, *P* = 0.00013. **d** Pearson correlation analysis results of enriched genes in two CRISPR library screen replicates. R=0.658, *P* <0.0001. **e-f** Dependency scores of HER2 and YAP for tumor cells across different cancer types. A score  < -1 indicates highly essential genes. **g** Immunohistochemical staining of YAP in GC patients from The Human Protein Atlas. **h** Transcriptional expression level of YAP between tumor and normal tissues in the TCGA-STAD cohort. Tumor number = 408, normal number = 211. * *P* <0.05 vs. normal. **i** Kaplan-Meier curves of time for probability showing the difference between HER2-positive GC patients with low and high YAP1 levels. n =202, log-rank test, *P* = 0.036.


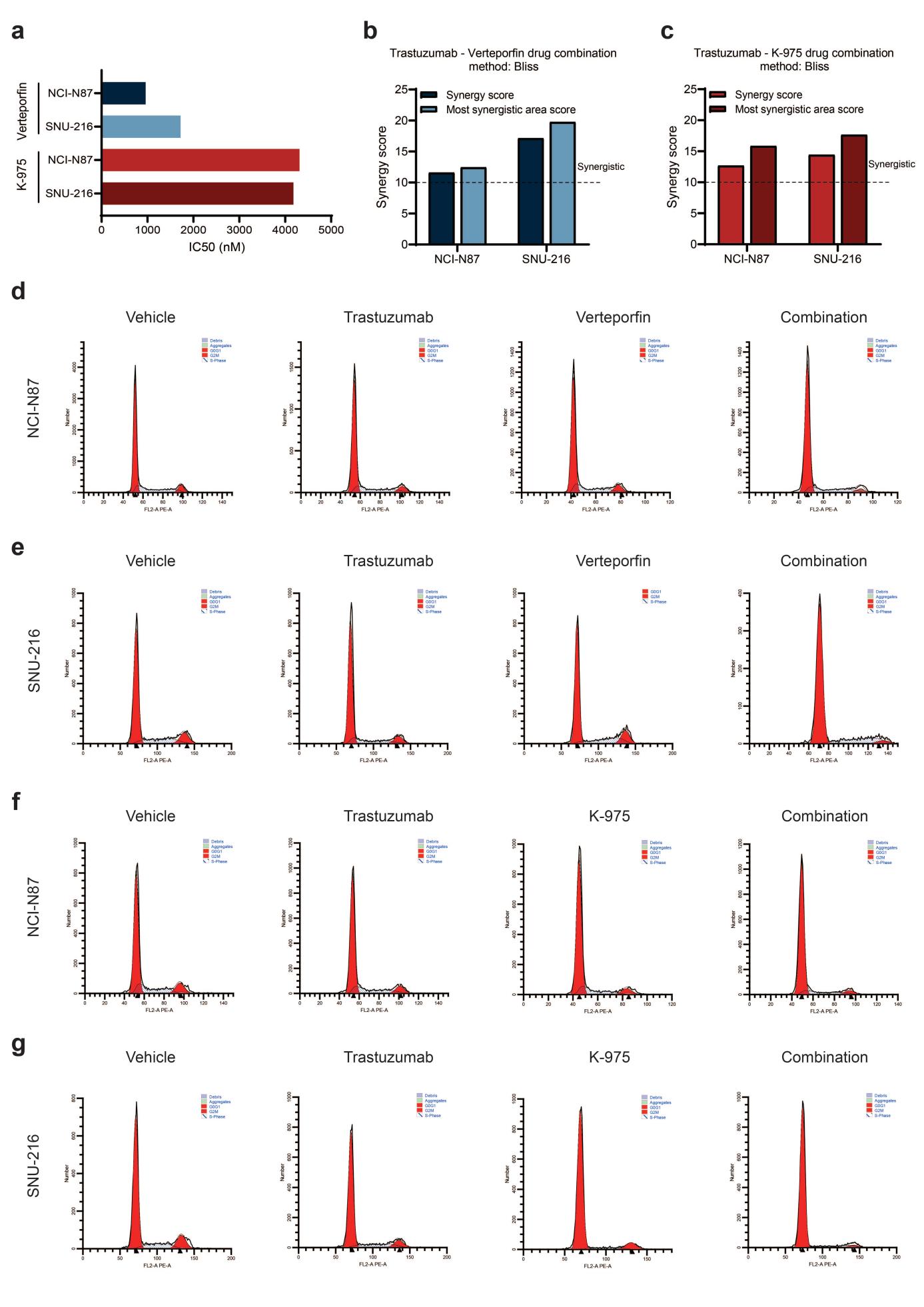


**Fig. S2.** **Synergistic model of trastuzumab and YAP inhibitors in HER2-positive GC cells. a** Average IC50 values generated from dose-response curves for verteporfin and K-975. **b-c** Bar plot of the synergy scores and most synergistic area scores of trastuzumab and verteporfin (left) or K-975 (right) drug combination among NCI-N87 and SNU-216 cells. A score > 10 indicates synergy. **d-e** Representative cell cycle distribution maps of NCI-N87 (upper) and SNU-216 (lower) cells treated with vehicle, trastuzumab (30 μg/ml), verteporfin (1 μM), or their combination for 48 h were determined by flow cytometry. **f-g** Representative cell cycle distribution maps of NCI-N87 (upper) and SNU-216 (lower) cells treated with vehicle, trastuzumab (30 μg/ml), K-975 (3 μM), or their combination for 48 h were determined by flow cytometry.


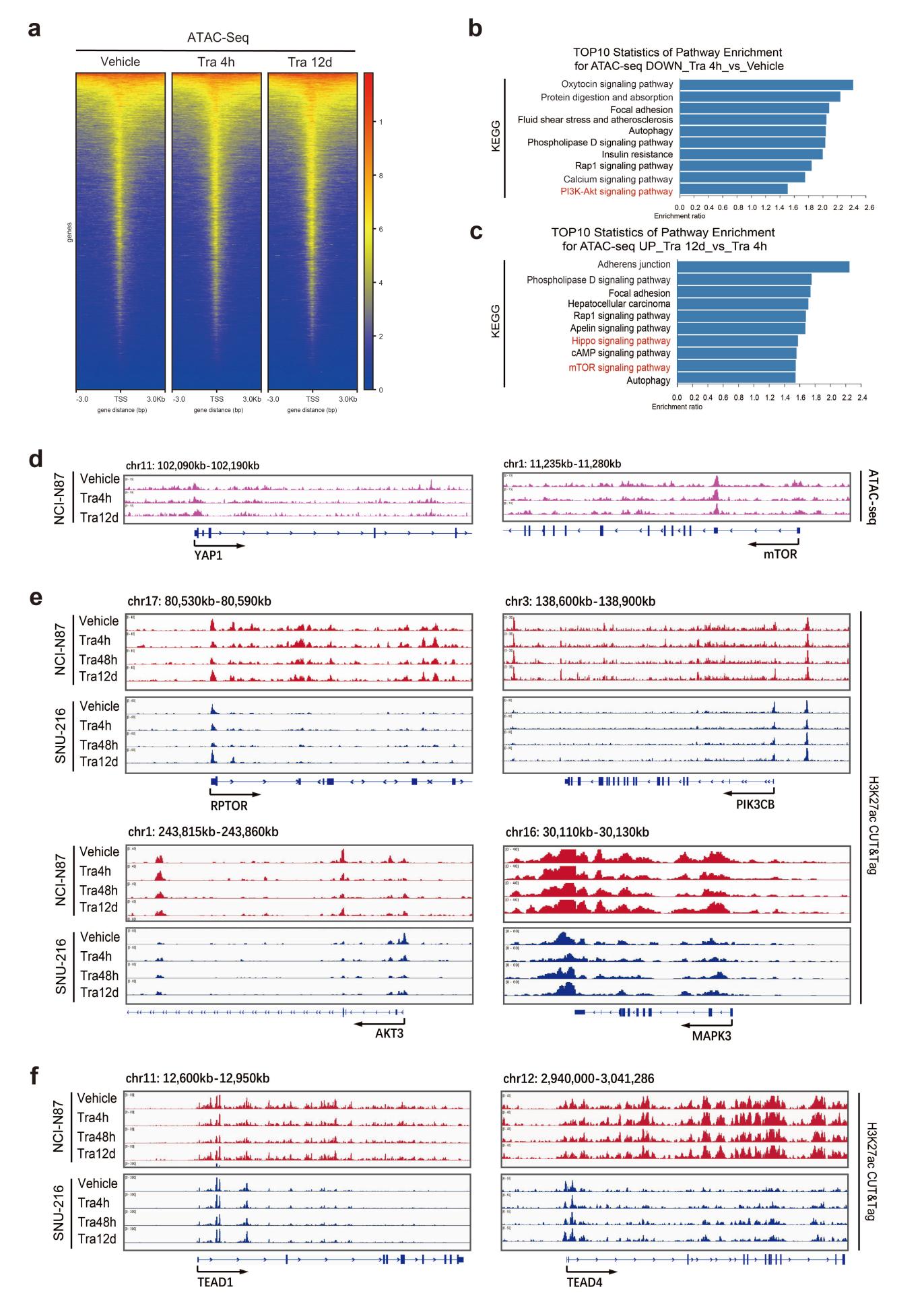


**Fig. S3. Prolonged trastuzumab treatment reprograms the Hippo and mTOR pathways. a** TSS heatmap of ATAC signals at ATAC-seq genes after 4h and 12 d of trastuzumab treatment of NCI-N87 cells. **b** The down-regulated ATAC-seq genes in NCI-N87 cells treated with trastuzumab vs. Vehcile for 4 h were subjected to enrichment analysis of KEGG pathways. **c** The up-regulated ATAC-seq genes in NCI-N87 cells treated with trastuzumab for 12 d vs. 4 h were subjected to enrichment analysis of KEGG pathways. **d** ATAC-seq tracks of YAP1and mTOR in NCI-N87 cells after vehicle treatment and 4 h or 12 d of trastuzumab treatment. **e** Gene tracks depicting H3K27ac signals at the RPTOR, PI3KCB, AKT3, and MAPK3 loci. The signal from trastuzumab at the indicated time points was normalized to that of the vehicle at the same time points. **f** CUT&Tag tracks H3K27ac signaling at the TEAD family (TEAD1/4) in the Hippo signaling pathway downstream of the YAP/TEAD transcription factor complex in NCI-N87 and SNU-216 cells upon vehicle treatment or after 4 h, 48 h, or 12 d of trastuzumab treatment.


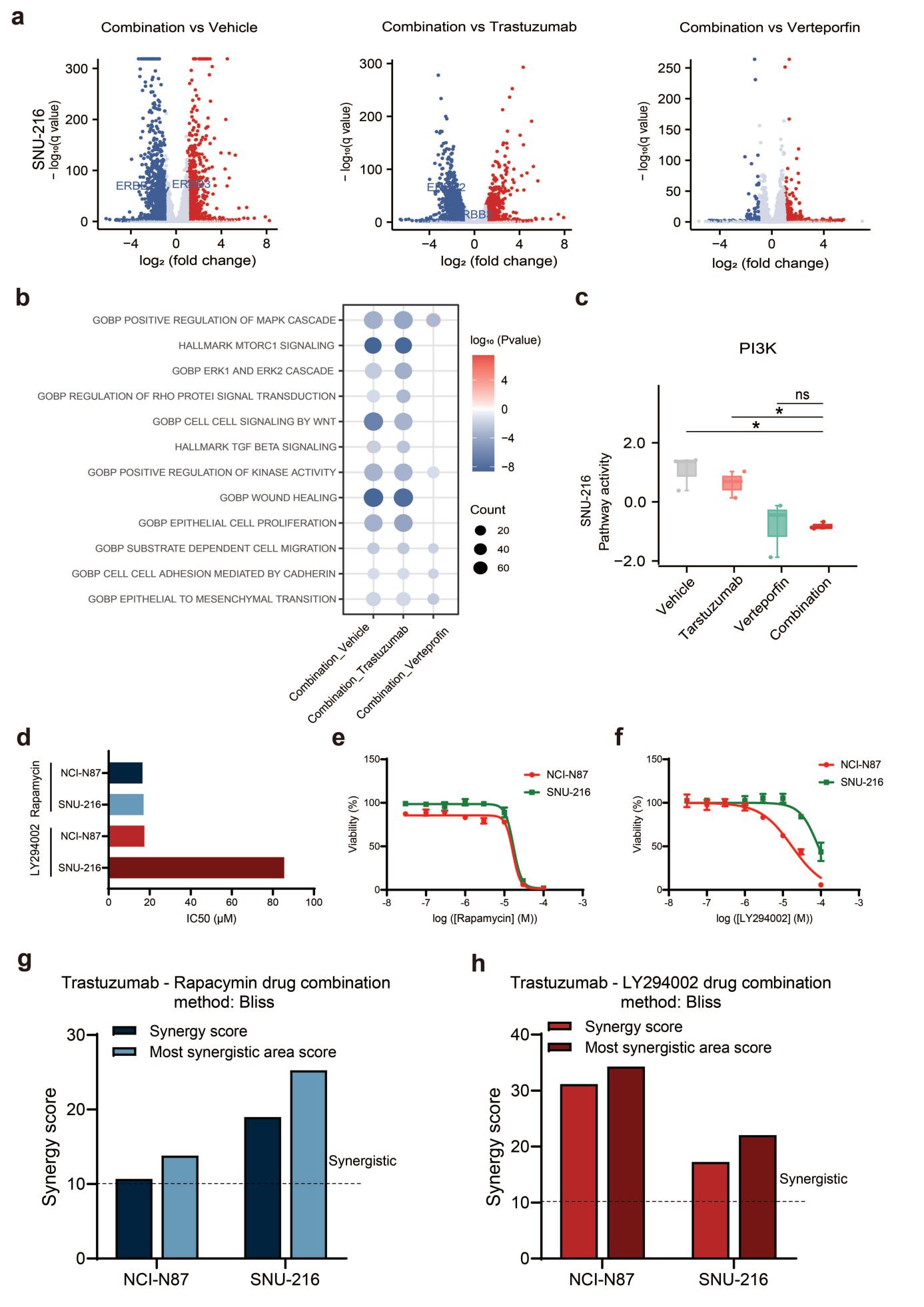


**Fig. S4. The addition of either a PI3K or mTOR inhibitor increased the efficacy of trastuzumab in HER2-positive GC cells. a** Volcano plot of −log10(q value) versus log2(fold change) of differentially expressed genes (DEGs) by 24h of trastuzumab treatment in SNU-216 cells. Genes increased are colored in red and genes decreased are colored in blue. Differential gene expression was normalized by DESeq, and adjusted P values were analyzed by Benjamini and Hochberg's approach. **b** Dot plot of representative enriched pathways in different comparisons of SNU-216 cells. Pathways enriched in up-regulated genes are colored in red and downregulated genes are in blue. **c** Box plots showing the activity levels of the PI3K signaling in the four cohorts of SNU-216 cells. **d** Average IC50 values generated from dose-response curves for the mTOR inhibitor (rapamycin) and PI3K inhibitor (LY294002). **e-f** The viability of NCI-N87 and SNU-216 cells was detected by CCK-8 assays after treatment with rapamycin (0-100 μM, left) or LY294002 (0-100 μM, right) treatment for 72 h. **g-h** Bar plot of the synergy score and most synergistic area score of combinations of trastuzumab and rapamycin (left) or LY294002 (right) among NCI-N87 and SNU-216 cells. A score > 10 indicates synergy.


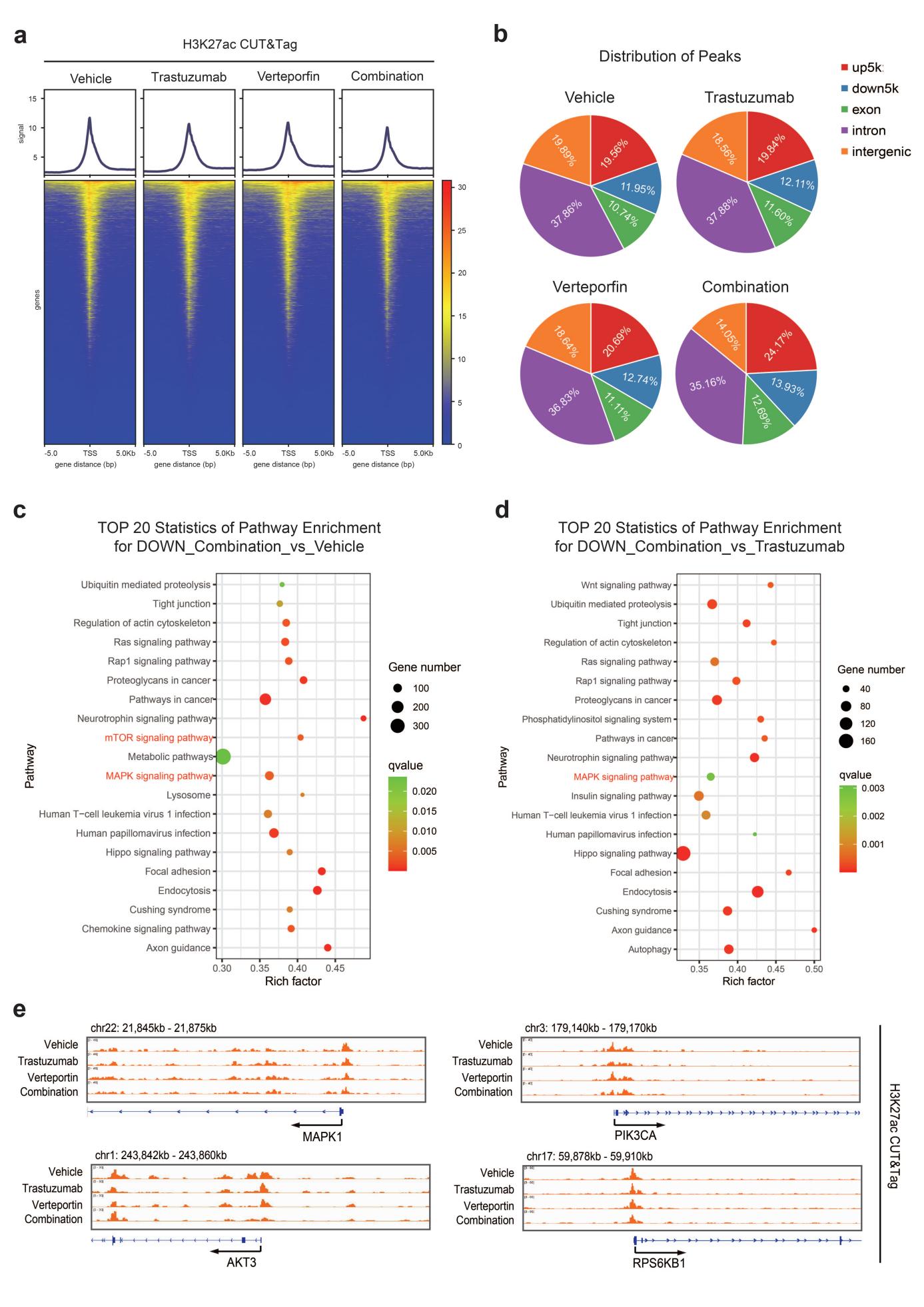


Fig. S5. H3K27ac CUT&Tag analysis demonstrated the downregulation of the ERK/mTOR and AKT/mTOR signaling pathways in the combination group of HER2-positive GC cells. a TSS heatmap showing that the H3K27ac signal decreased after 24 h of combination treatment compared to that of vehicle or monotherapy. b Pie plot of the genomic distribution of peaks in NCI-N87 cells treated with vehicle, trastuzumab, verteprofin, or their combination for 24 h. c-d Statistics for the top 20 KEGG pathway analyses of the decreased H3K27ac binding peaks at candidate target genes of Combination_vs_Vehicle (left) or DOWN_Combination_vs_Trastuzumab (right). e CUT&Tag tracks H3K27ac signaling at MAPK1, PIK3CA, AKT3, and RPS6KB1 in NCI-N87 cells upon four cohorts of 24 h.

**Table S1. Panel of 7 GC cell lines used in the study**

| **Name** | **Median** | **sex** | **source** | **Collection site** | **Primary or metastasis** | **Mycoplasma** |
| --- | --- | --- | --- | --- | --- | --- |
| SGC-7901 | RPMI + 10% FBS | Female | CSIC | Lymph node | Metastasis | Negative |
| MGC-803 | DMEM + 10% FBS | Male | CSIC | stomach | Primary | Negative |
| HGC-27 | RPMI + 10% FBS | Female | ECACC  ([94042256](https://www.culturecollections.org.uk/products/celllines/generalcell/detail.jsp?refId=94042256&collection=ecacc_gc)) | Lymph node | Metastasis | Negative |
| NCI-N87 | RPMI + 10% FBS | Male | ATCC  (CRL-5822) | liver | Metastasis | Negative |
| AGS | RPMI + 10% FBS | Female | ATCC  (CRL-1739) | stomach | Primary | Negative |
| MKN45 | RPMI + 10% FBS | Female | CSIC | liver | Metastasis | Negative |
| SNU-216 | RPMI + 10% FBS | Female | CTCC  (CTCC-007-0397) | Lymph node | Metastasis | Negative |
