# Supplementary material for: YAP inhibition overcomes adaptive resistance in HER2-positive gastric cancer treated with trastuzumab via the AKT/mTOR and ERK/mTOR axis
Source: Gastric Cancer. 2024 May 23;27(4):785–801. doi: 10.1007/s10120-024-01508-3 (PMC11193831; doi:10.1007/s10120-024-01508-3)
Supplement: Supplementary file 5 — (PDF 2516 kb) [file 10120_2024_1508_MOESM5_ESM.pdf]

Fig. 1a

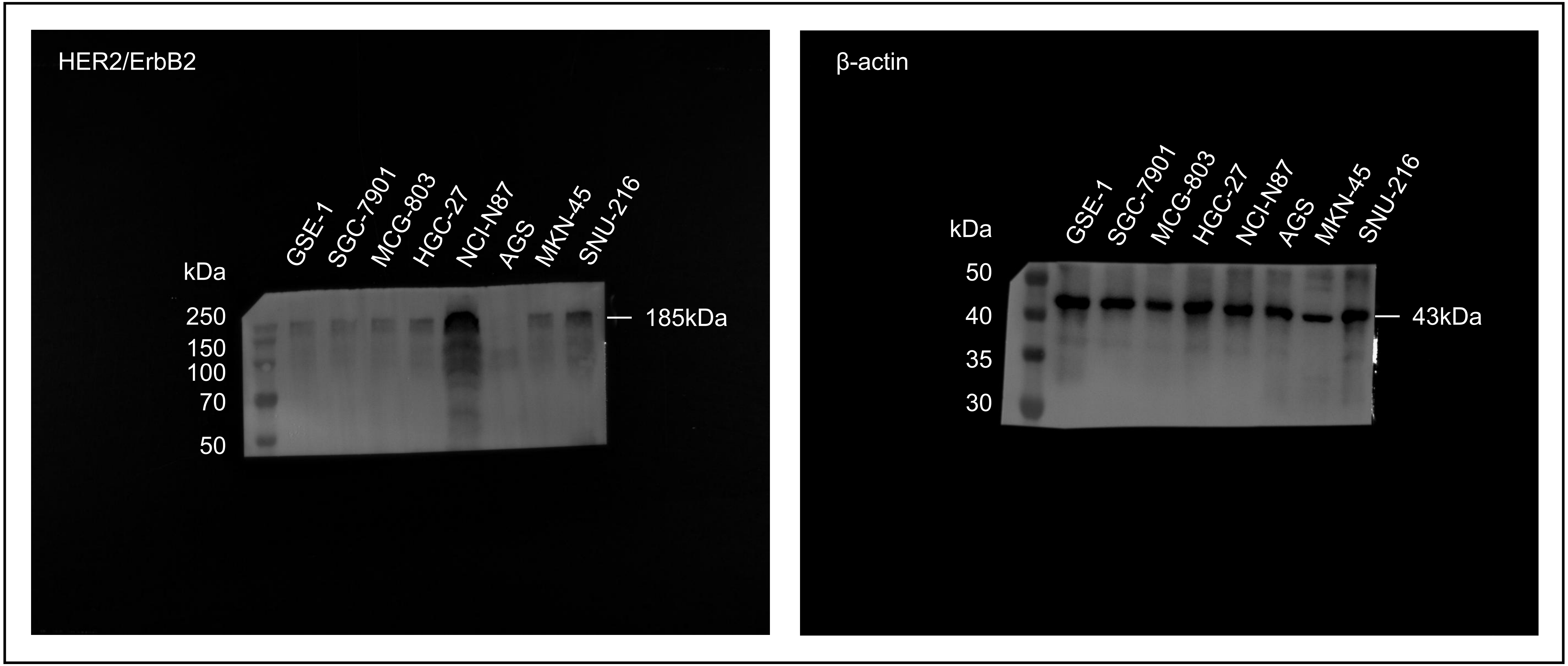
HER2/ErbB2
GSE-1
SGC-7901
MCG-803
HGC-27
NCI-N87
AGS
MKN-45
SNU-216
kDa
250
150
100
70
50
185kDa
β-actin
GSE-1
SGC-7901
MCG-803
HGC-27
NCI-N87
AGS
MKN-45
SNU-216
kDa
50
40
35
30
43kDa

Fig. 1g

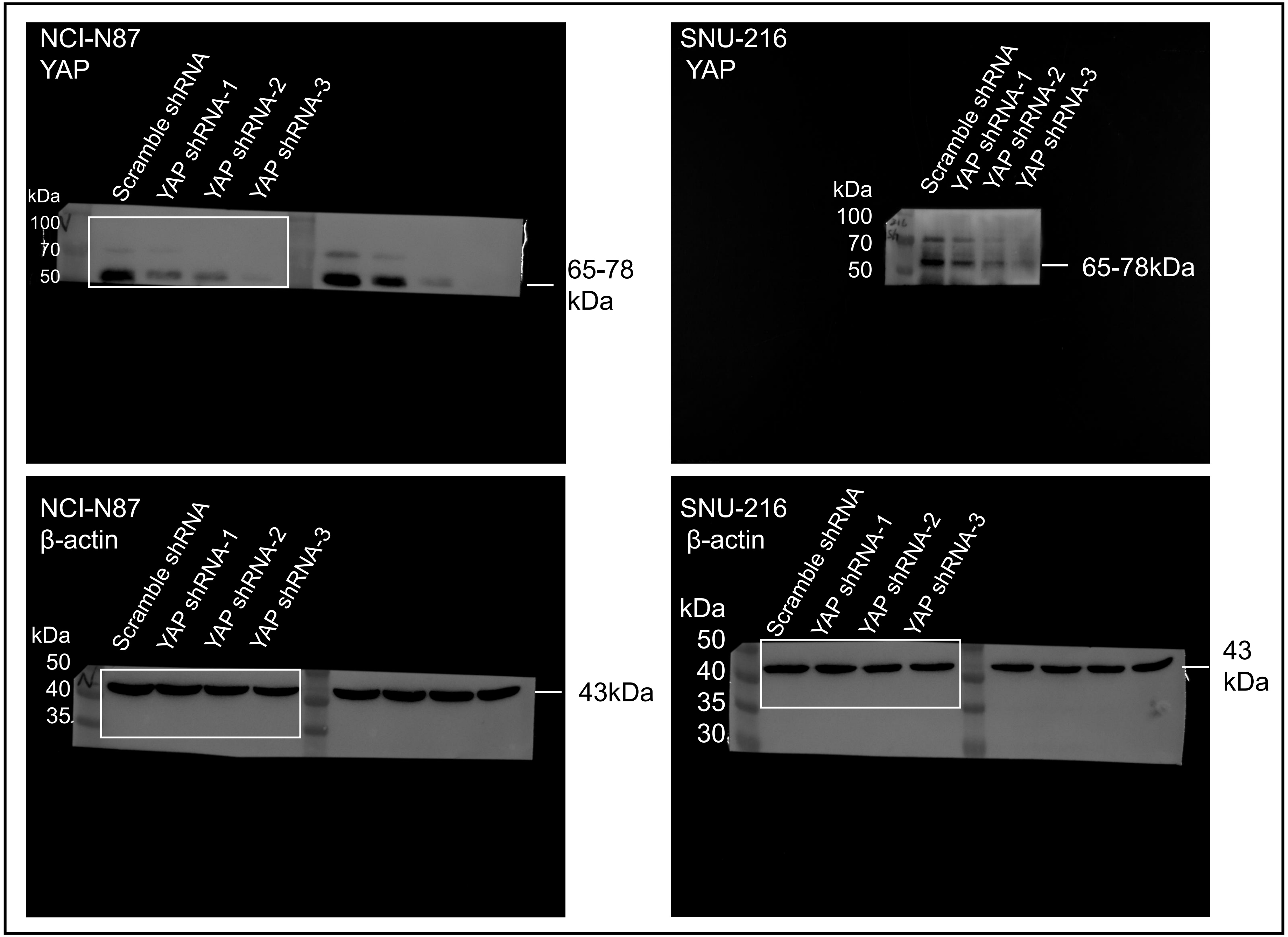
NCI-N87
YAP
Scramble shRNA
YAP shRNA-1
YAP shRNA-2
YAP shRNA-3
kDa
100
70
50
65-78
kDa
SNU-216
YAP
Scramble shRNA
YAP shRNA-1
YAP shRNA-2
YAP shRNA-3
kDa
100
70
50
65-78kDa
NCI-N87
β-actin
Scramble shRNA
YAP shRNA-1
YAP shRNA-2
YAP shRNA-3
kDa
50
40
35
43kDa
SNU-216
β-actin
Scramble shRNA
YAP shRNA-1
YAP shRNA-2
YAP shRNA-3
kDa
50
40
35
30
43
kDa

Fig. 2a

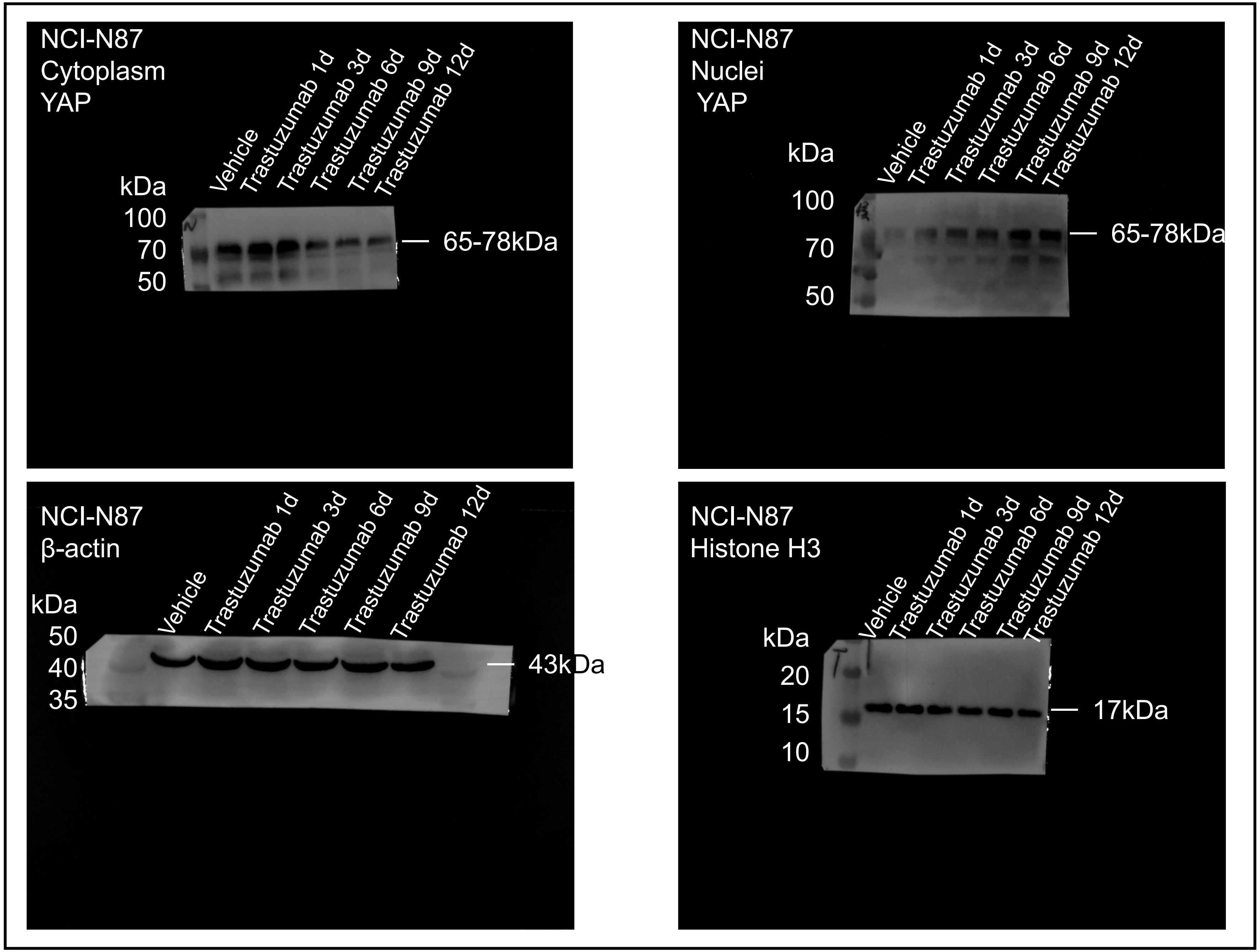
NCI-N87
Cytoplasm
YAP
Vehicle
Trastuzumab 1d
Trastuzumab 3d
Trastuzumab 6d
Trastuzumab 9d
Trastuzumab 12d
kDa
100
70
50
65-78kDa
NCI-N87
Nuclei
YAP
kDa
100
70
50
65-78kDa
NCI-N87
β-actin
kDa
50
40
35
43kDa
NCI-N87
Histone H3
kDa
20
15
10
17kDa

Fig. 2a

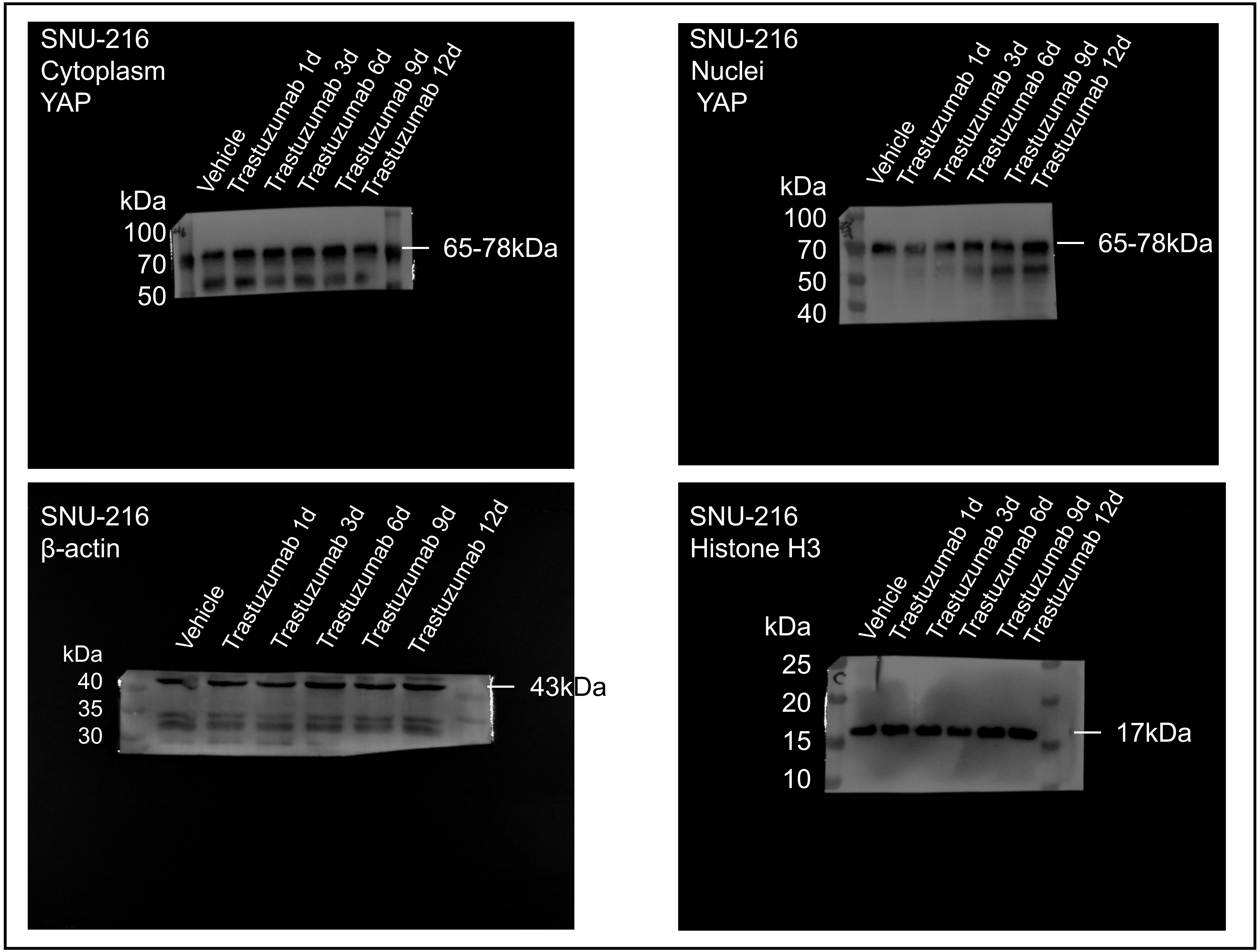
SNU-216
Cytoplasm
YAP
Vehicle
Trastuzumab 1d
Trastuzumab 3d
Trastuzumab 6d
Trastuzumab 9d
Trastuzumab 12d
kDa
100
70
50
65-78kDa
SNU-216
Nuclei
YAP
Vehicle
Trastuzumab 1d
Trastuzumab 3d
Trastuzumab 6d
Trastuzumab 9d
Trastuzumab 12d
kDa
100
70
50
40
65-78kDa
SNU-216
β-actin
Vehicle
Trastuzumab 1d
Trastuzumab 3d
Trastuzumab 6d
Trastuzumab 9d
Trastuzumab 12d
kDa
40
35
30
43kDa
SNU-216
Histone H3
Vehicle
Trastuzumab 1d
Trastuzumab 3d
Trastuzumab 6d
Trastuzumab 9d
Trastuzumab 12d
kDa
25
20
15
10
17kDa

Fig. 3g

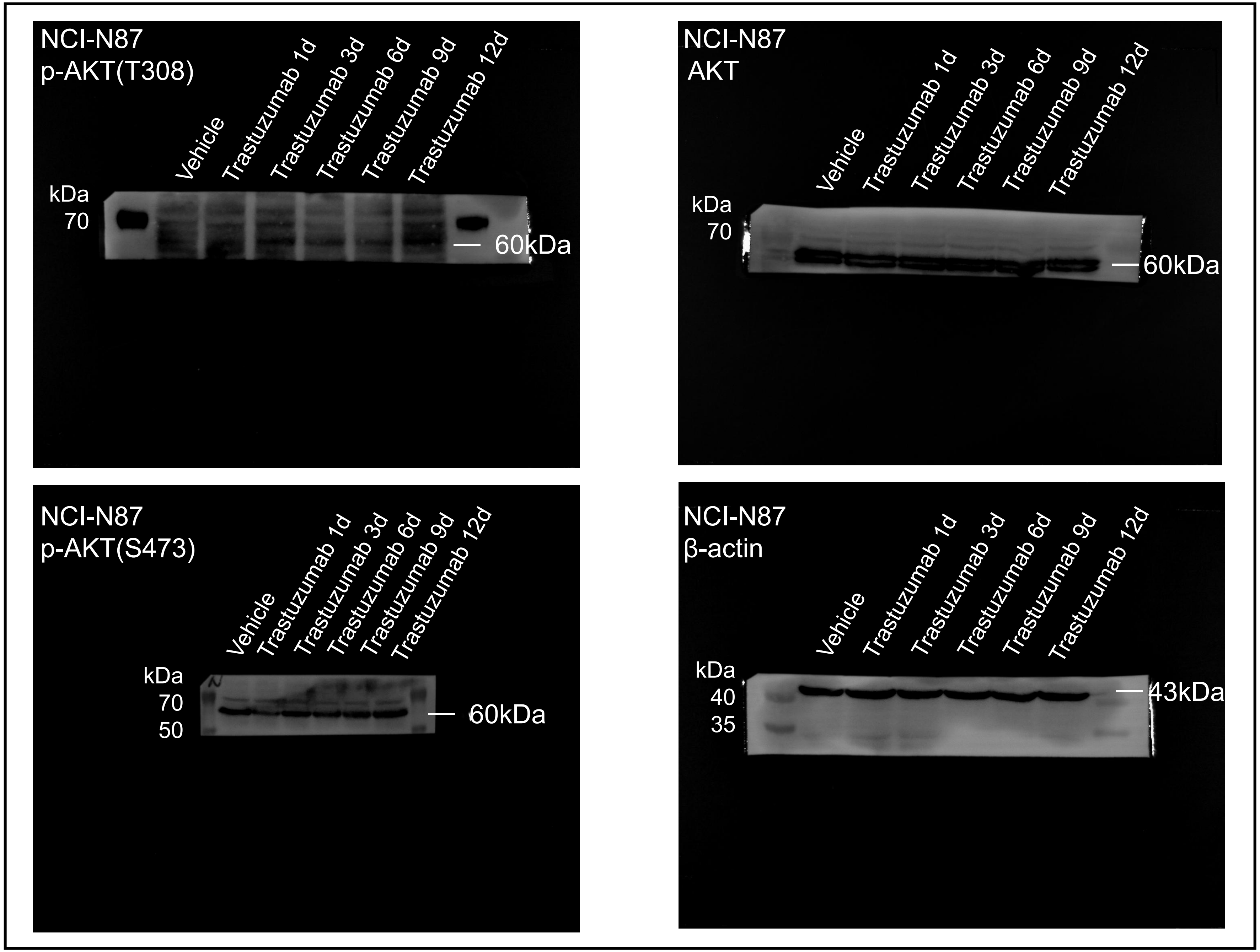

NCI-N87
p-AKT(T308)
Vehicle
Trastuzumab 1d
Trastuzumab 3d
Trastuzumab 6d
Trastuzumab 9d
Trastuzumab 12d
kDa
70
60kDa
NCI-N87
AKT
Vehicle
Trastuzumab 1d
Trastuzumab 3d
Trastuzumab 6d
Trastuzumab 9d
Trastuzumab 12d
kDa
70
60kDa
NCI-N87
p-AKT(S473)
Vehicle
Trastuzumab 1d
Trastuzumab 3d
Trastuzumab 6d
Trastuzumab 9d
Trastuzumab 12d
kDa
70
50
60kDa
NCI-N87
β-actin
Vehicle
Trastuzumab 1d
Trastuzumab 3d
Trastuzumab 6d
Trastuzumab 9d
Trastuzumab 12d
kDa
40
35
43kDa

Fig. 3g

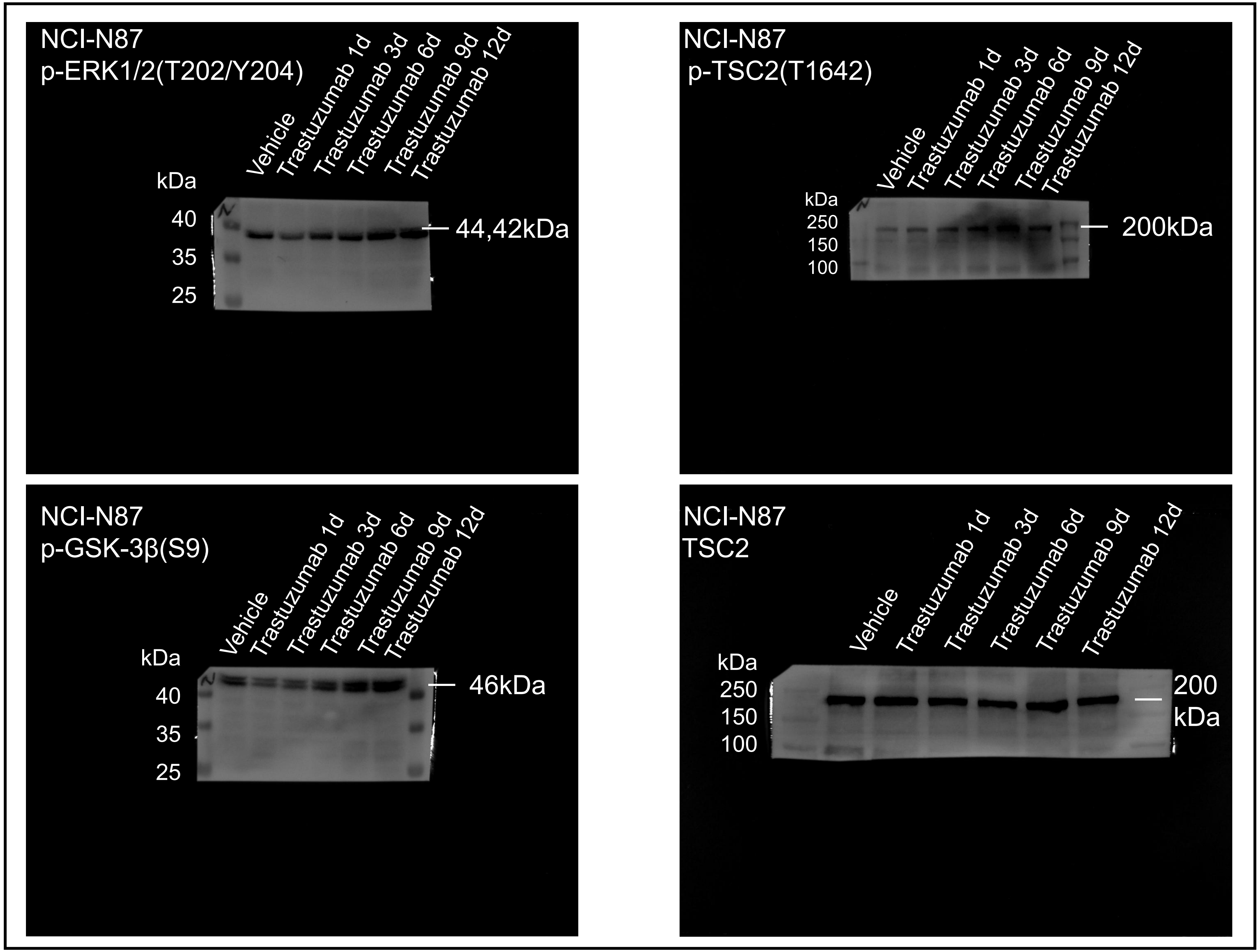
NCI-N87
p-ERK1/2(T202/Y204)
Vehicle
Trastuzumab 1d
Trastuzumab 3d
Trastuzumab 6d
Trastuzumab 9d
Trastuzumab 12d
kDa
40
35
25
44,42kDa
NCI-N87
p-TSC2(T1642)
Vehicle
Trastuzumab 1d
Trastuzumab 3d
Trastuzumab 6d
Trastuzumab 9d
Trastuzumab 12d
kDa
250
150
100
200kDa
NCI-N87
p-GSK-3β(S9)
Vehicle
Trastuzumab 1d
Trastuzumab 3d
Trastuzumab 6d
Trastuzumab 9d
Trastuzumab 12d
kDa
40
35
25
46kDa
NCI-N87
TSC2
Vehicle
Trastuzumab 1d
Trastuzumab 3d
Trastuzumab 6d
Trastuzumab 9d
Trastuzumab 12d
kDa
250
150
100
200 kDa

Fig. 3g

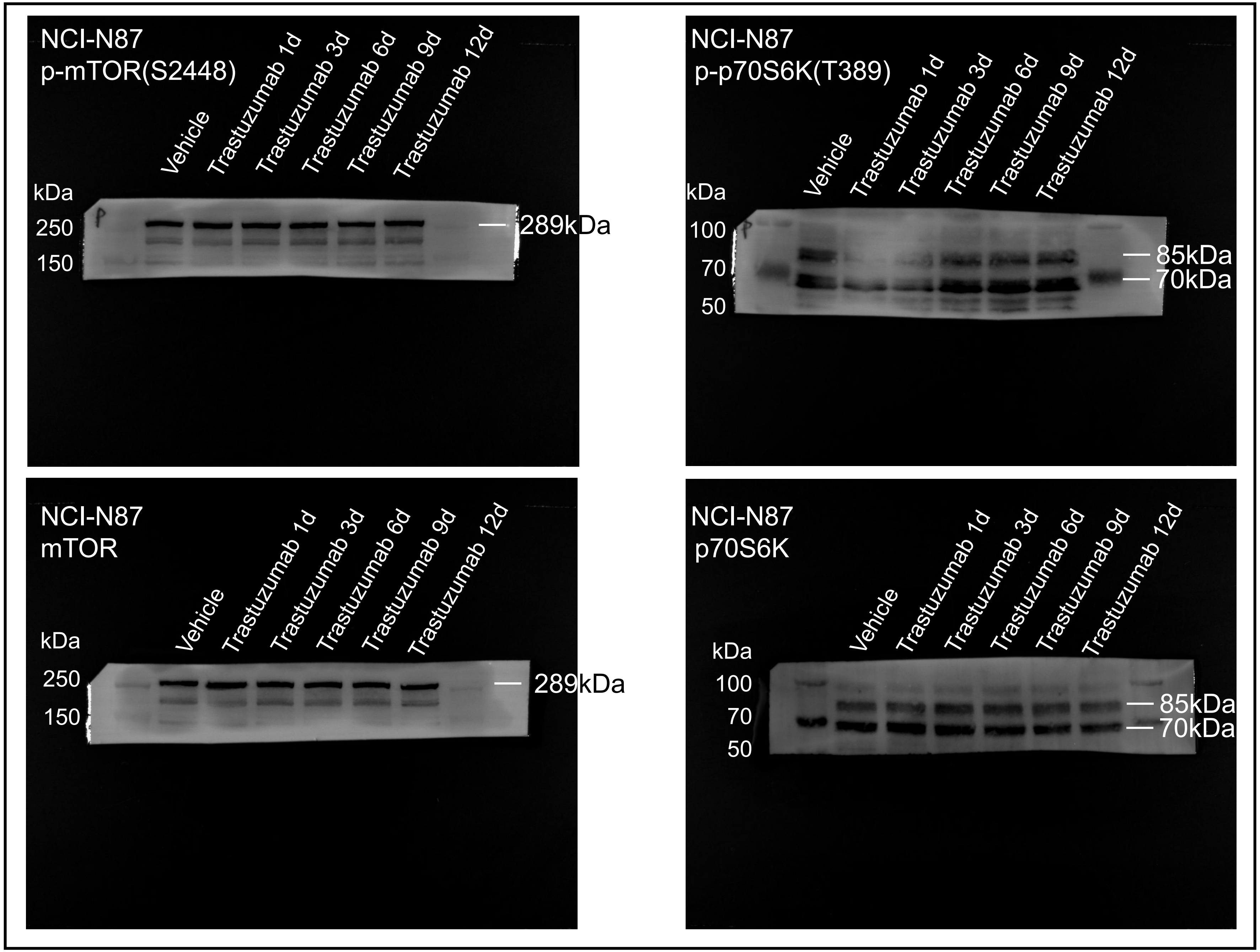
NCI-N87
p-mTOR(S2448)
Vehicle
Trastuzumab 1d
Trastuzumab 3d
Trastuzumab 6d
Trastuzumab 9d
Trastuzumab 12d
kDa
250
150
289kDa
NCI-N87
p-p70S6K(T389)
Vehicle
Trastuzumab 1d
Trastuzumab 3d
Trastuzumab 6d
Trastuzumab 9d
Trastuzumab 12d
kDa
100
70
50
85kDa
70kDa
NCI-N87
mTOR
Vehicle
Trastuzumab 1d
Trastuzumab 3d
Trastuzumab 6d
Trastuzumab 9d
Trastuzumab 12d
kDa
250
150
289kDa
NCI-N87
p70S6K
Vehicle
Trastuzumab 1d
Trastuzumab 3d
Trastuzumab 6d
Trastuzumab 9d
Trastuzumab 12d
kDa
100
70
50
85kDa
70kDa

Fig. 3g

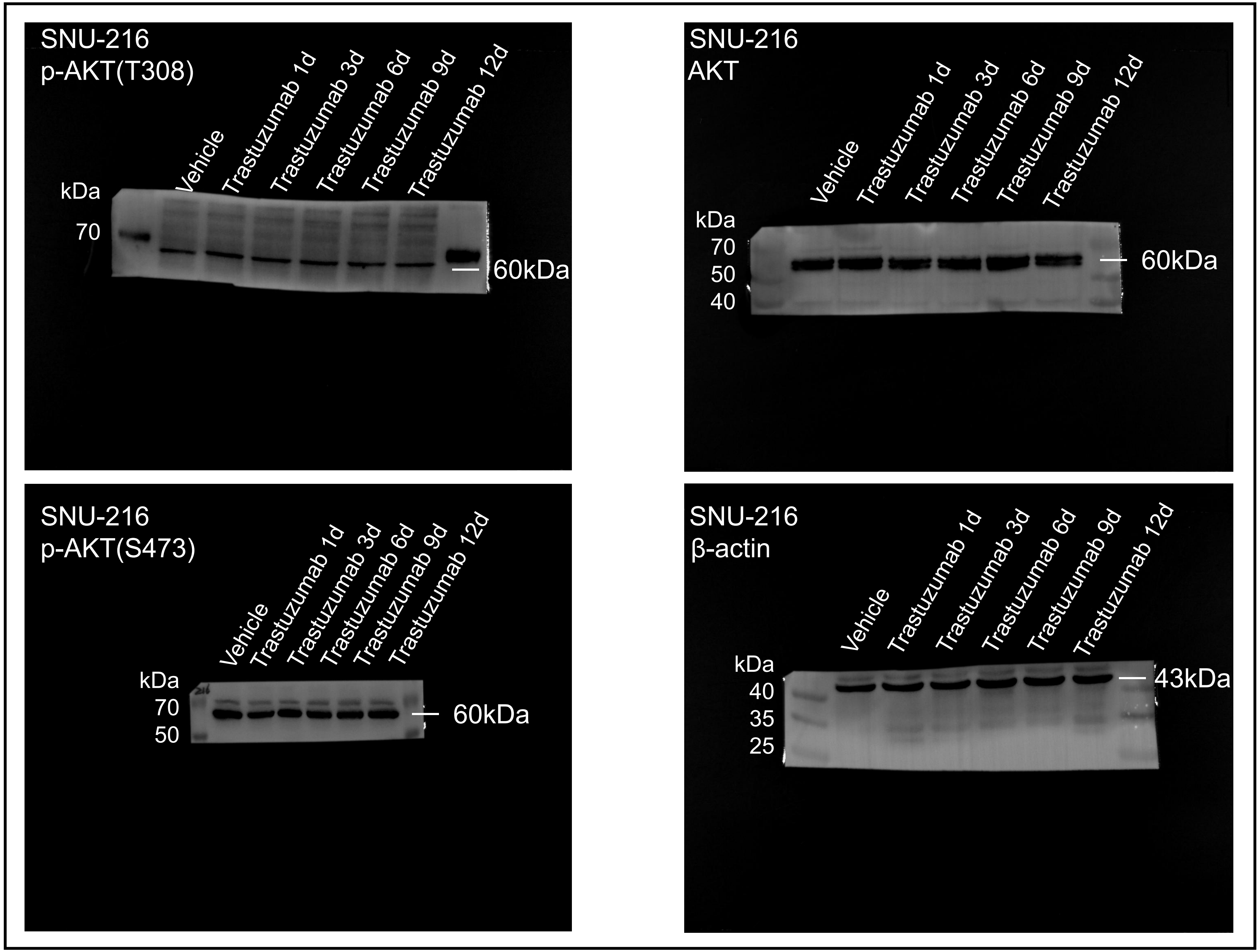
SNU-216
p-AKT(T308)
Vehicle
Trastuzumab 1d
Trastuzumab 3d
Trastuzumab 6d
Trastuzumab 9d
Trastuzumab 12d
kDa
70
60kDa
SNU-216
AKT
Vehicle
Trastuzumab 1d
Trastuzumab 3d
Trastuzumab 6d
Trastuzumab 9d
Trastuzumab 12d
kDa
70
50
40
60kDa
SNU-216
p-AKT(S473)
Vehicle
Trastuzumab 1d
Trastuzumab 3d
Trastuzumab 6d
Trastuzumab 9d
Trastuzumab 12d
kDa
70
50
60kDa
SNU-216
β-actin
Vehicle
Trastuzumab 1d
Trastuzumab 3d
Trastuzumab 6d
Trastuzumab 9d
Trastuzumab 12d
kDa
40
35
25
43kDa

Fig. 3g

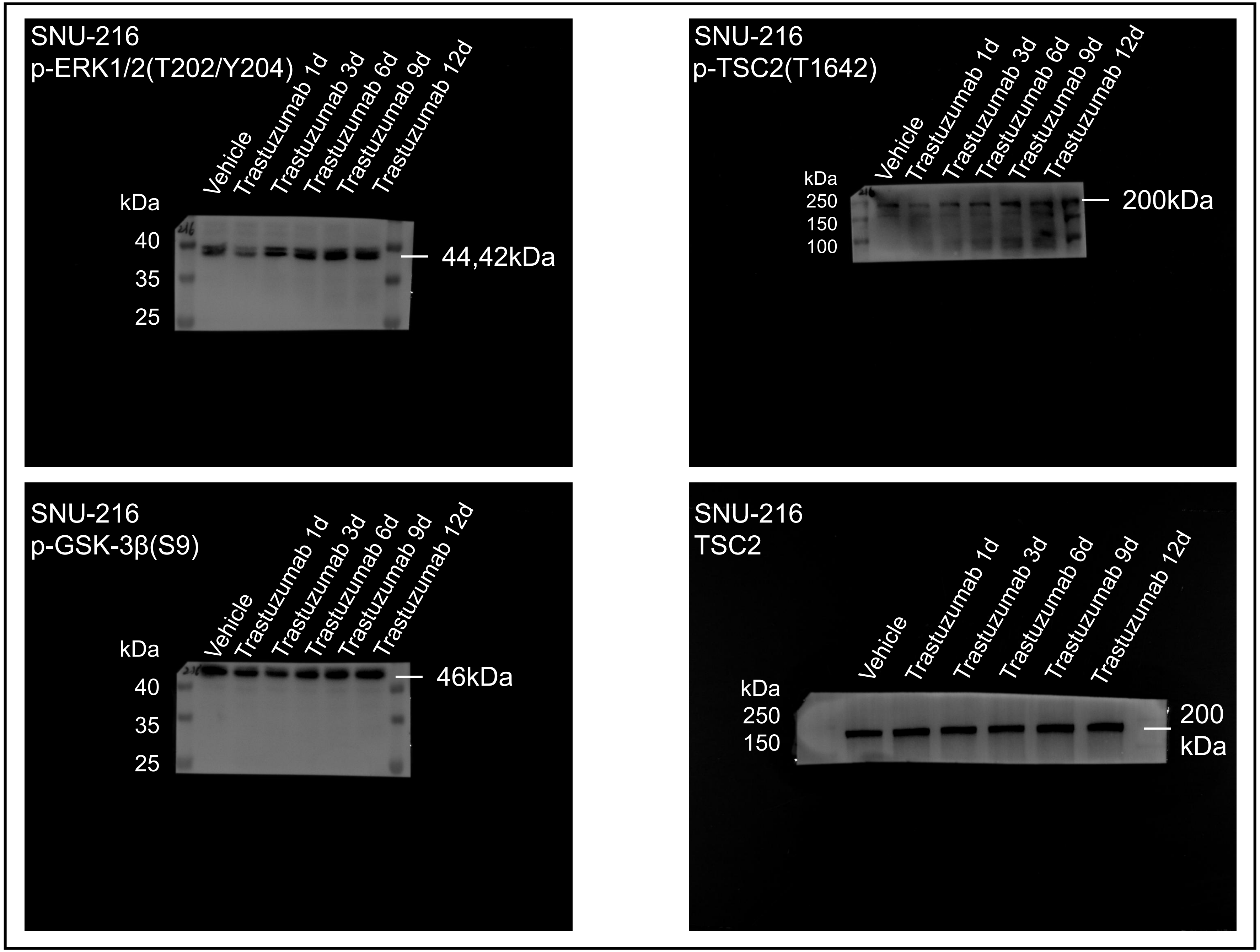
SNU-216
p-ERK1/2(T202/Y204)
Vehicle
Trastuzumab 1d
Trastuzumab 3d
Trastuzumab 6d
Trastuzumab 9d
Trastuzumab 12d
kDa
40
35
25
44,42kDa
SNU-216
p-TSC2(T1642)
Vehicle
Trastuzumab 1d
Trastuzumab 3d
Trastuzumab 6d
Trastuzumab 9d
Trastuzumab 12d
kDa
250
150
100
200kDa
SNU-216
p-GSK-3β(S9)
Vehicle
Trastuzumab 1d
Trastuzumab 3d
Trastuzumab 6d
Trastuzumab 9d
Trastuzumab 12d
kDa
40
35
25
46kDa
SNU-216
TSC2
Vehicle
Trastuzumab 1d
Trastuzumab 3d
Trastuzumab 6d
Trastuzumab 9d
Trastuzumab 12d
kDa
250
150
200
kDa

Fig. 3g

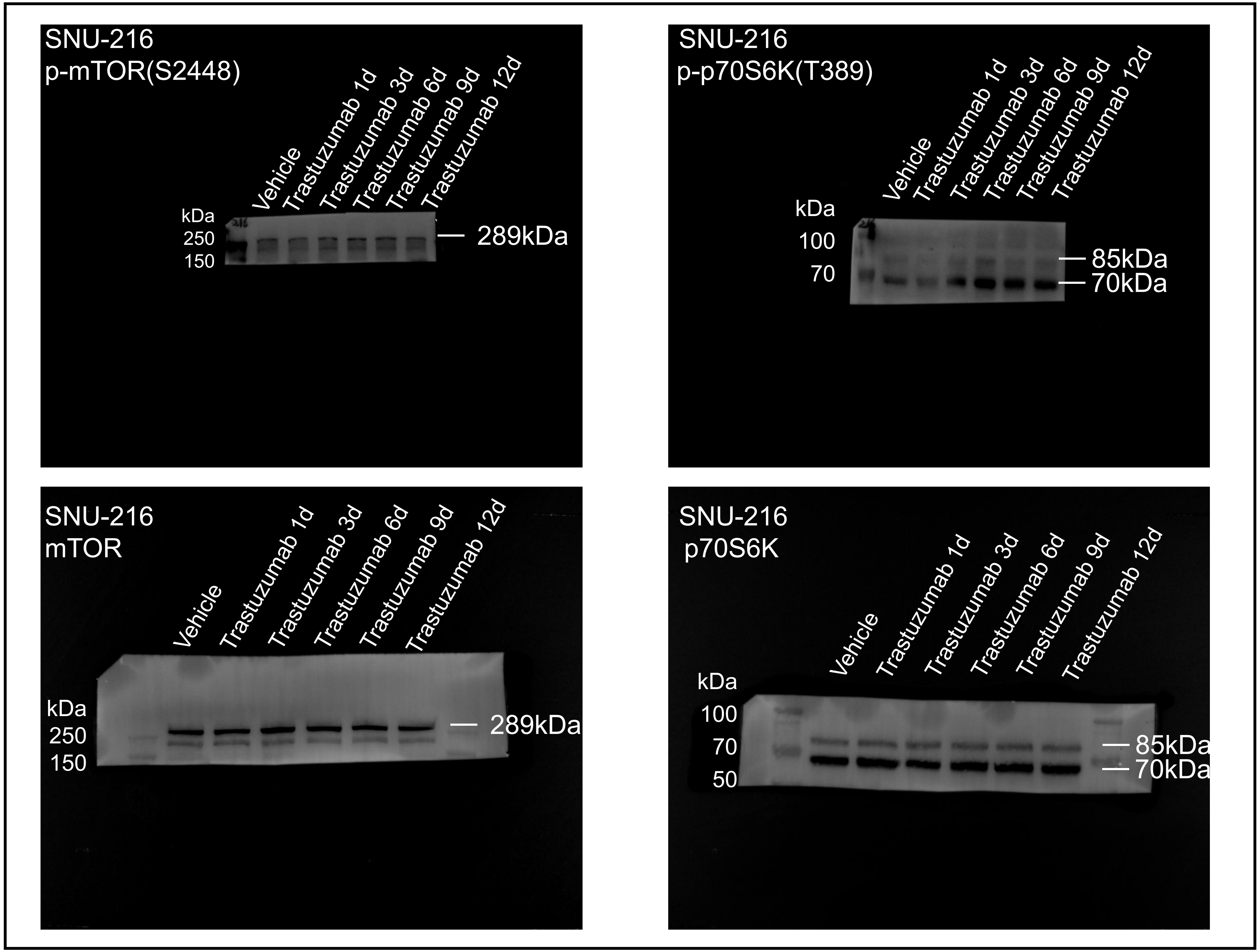
SNU-216
p-mTOR(S2448)
Vehicle
Trastuzumab 1d
Trastuzumab 3d
Trastuzumab 6d
Trastuzumab 9d
Trastuzumab 12d
kDa
250
150
289kDa
SNU-216
p-p70S6K(T389)
Vehicle
Trastuzumab 1d
Trastuzumab 3d
Trastuzumab 6d
Trastuzumab 9d
Trastuzumab 12d
kDa
100
70
85kDa
70kDa
SNU-216
mTOR
Vehicle
Trastuzumab 1d
Trastuzumab 3d
Trastuzumab 6d
Trastuzumab 9d
Trastuzumab 12d
kDa
250
150
289kDa
SNU-216
p70S6K
Vehicle
Trastuzumab 1d
Trastuzumab 3d
Trastuzumab 6d
Trastuzumab 9d
Trastuzumab 12d
kDa
100
70
50
85kDa
70kDa

Fig. 4d

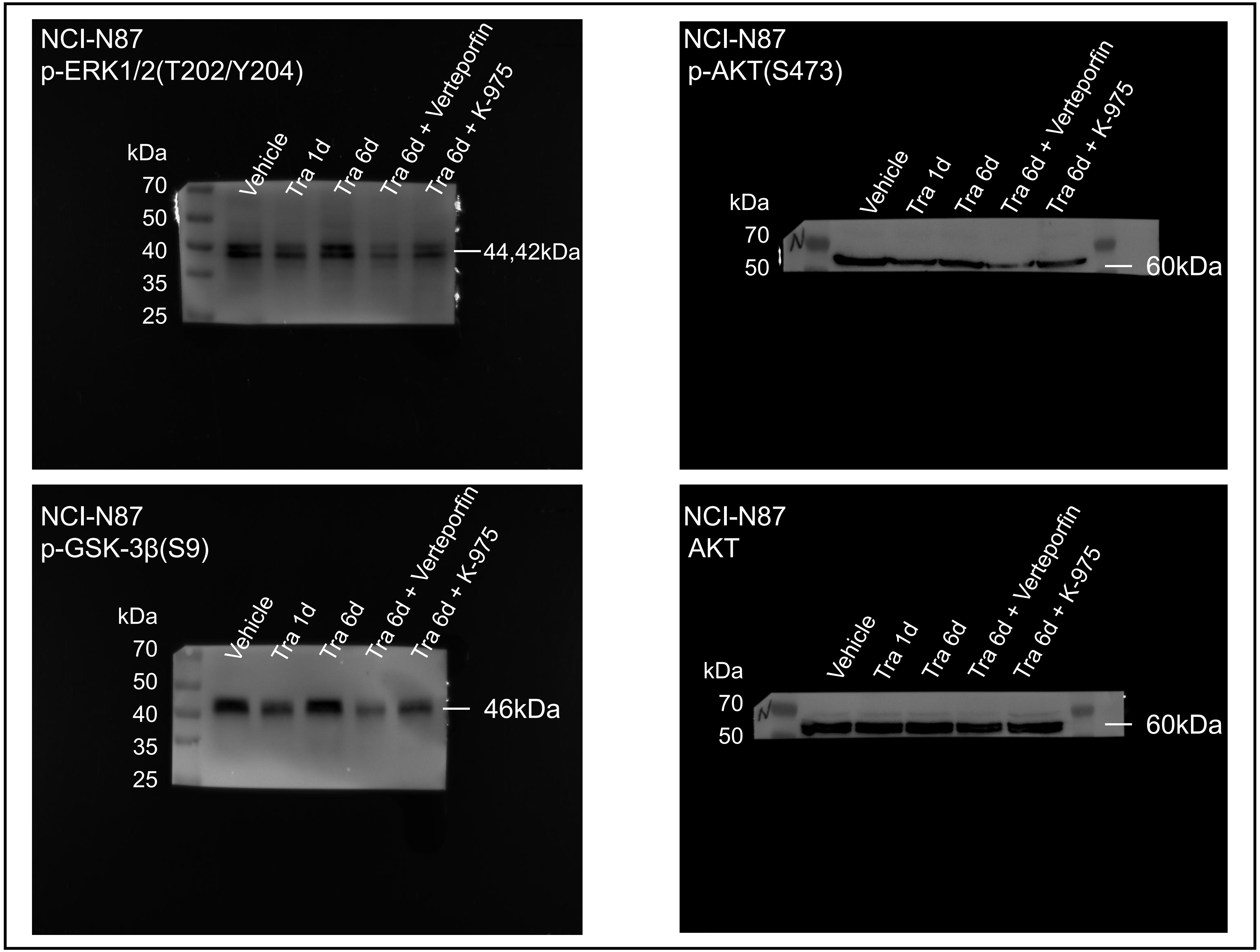
NCI-N87
p-ERK1/2(T202/Y204)
kDa
70
50
40
35
25
Vehicle
Tra 1d
Tra 6d
Tra 6d + Verteporfin
Tra 6d + K-975
44,42kDa
NCI-N87
p-AKT(S473)
kDa
70
50
Vehicle
Tra 1d
Tra 6d
Tra 6d + Verteporfin
Tra 6d + K-975
60kDa
NCI-N87
p-GSK-3β(S9)
kDa
70
50
40
35
25
Vehicle
Tra 1d
Tra 6d
Tra 6d + Verteporfin
Tra 6d + K-975
46kDa
NCI-N87
AKT
kDa
70
50
Vehicle
Tra 1d
Tra 6d
Tra 6d + Verteporfin
Tra 6d + K-975
60kDa

Fig. 4d

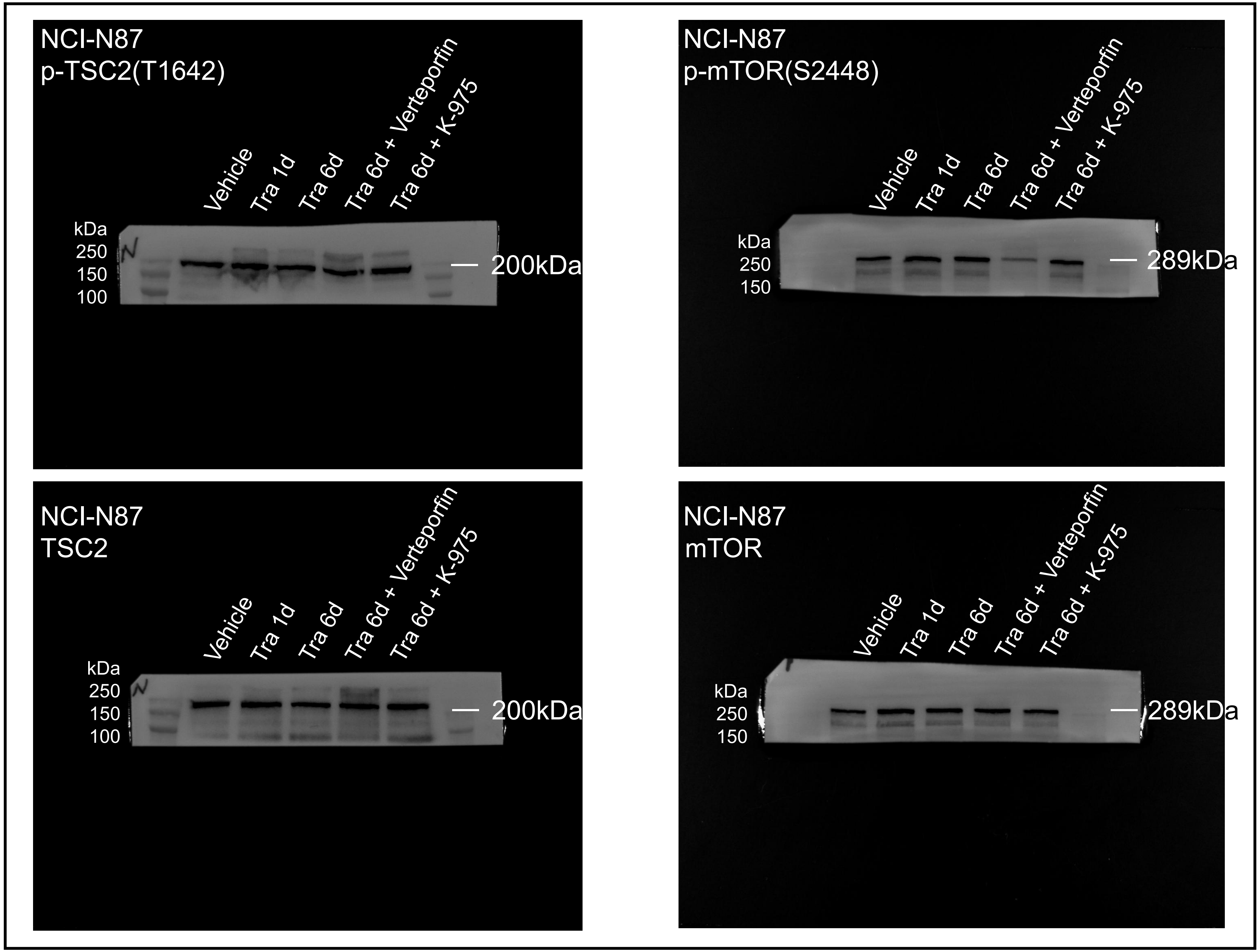

NCI-N87
p-TSC2(T1642)
Vehicle
Tra 1d
Tra 6d
Tra 6d + Verteporfin
Tra 6d + K-975
kDa
250
150
100
200kDa
NCI-N87
p-mTOR(S2448)
Vehicle
Tra 1d
Tra 6d
Tra 6d + Verteporfin
Tra 6d + K-975
kDa
250
150
289kDa
NCI-N87
TSC2
Vehicle
Tra 1d
Tra 6d
Tra 6d + Verteporfin
Tra 6d + K-975
kDa
250
150
100
200kDa
NCI-N87
mTOR
Vehicle
Tra 1d
Tra 6d
Tra 6d + Verteporfin
Tra 6d + K-975
kDa
250
150
289kDa

Fig. 4d

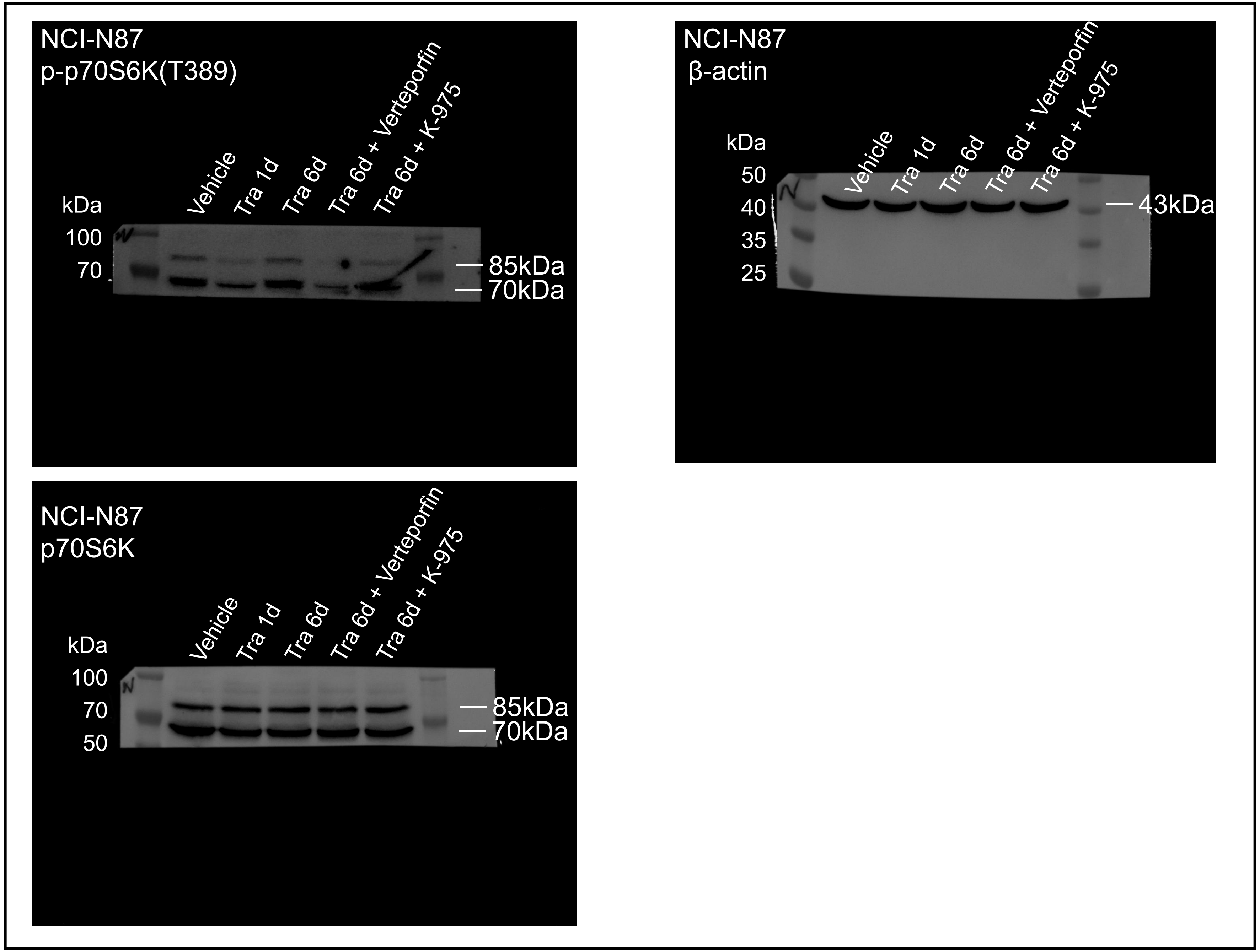
NCI-N87
p-p70S6K(T389)
kDa
100
70
Vehicle
Tra 1d
Tra 6d
Tra 6d + Verteporfin
Tra 6d + K-975
85kDa
70kDa
NCI-N87
β-actin
kDa
50
40
35
25
Vehicle
Tra 1d
Tra 6d
Tra 6d + Verteporfin
Tra 6d + K-975
43kDa
NCI-N87
p70S6K
kDa
100
70
50
Vehicle
Tra 1d
Tra 6d
Tra 6d + Verteporfin
Tra 6d + K-975
85kDa
70kDa

Fig. 4d

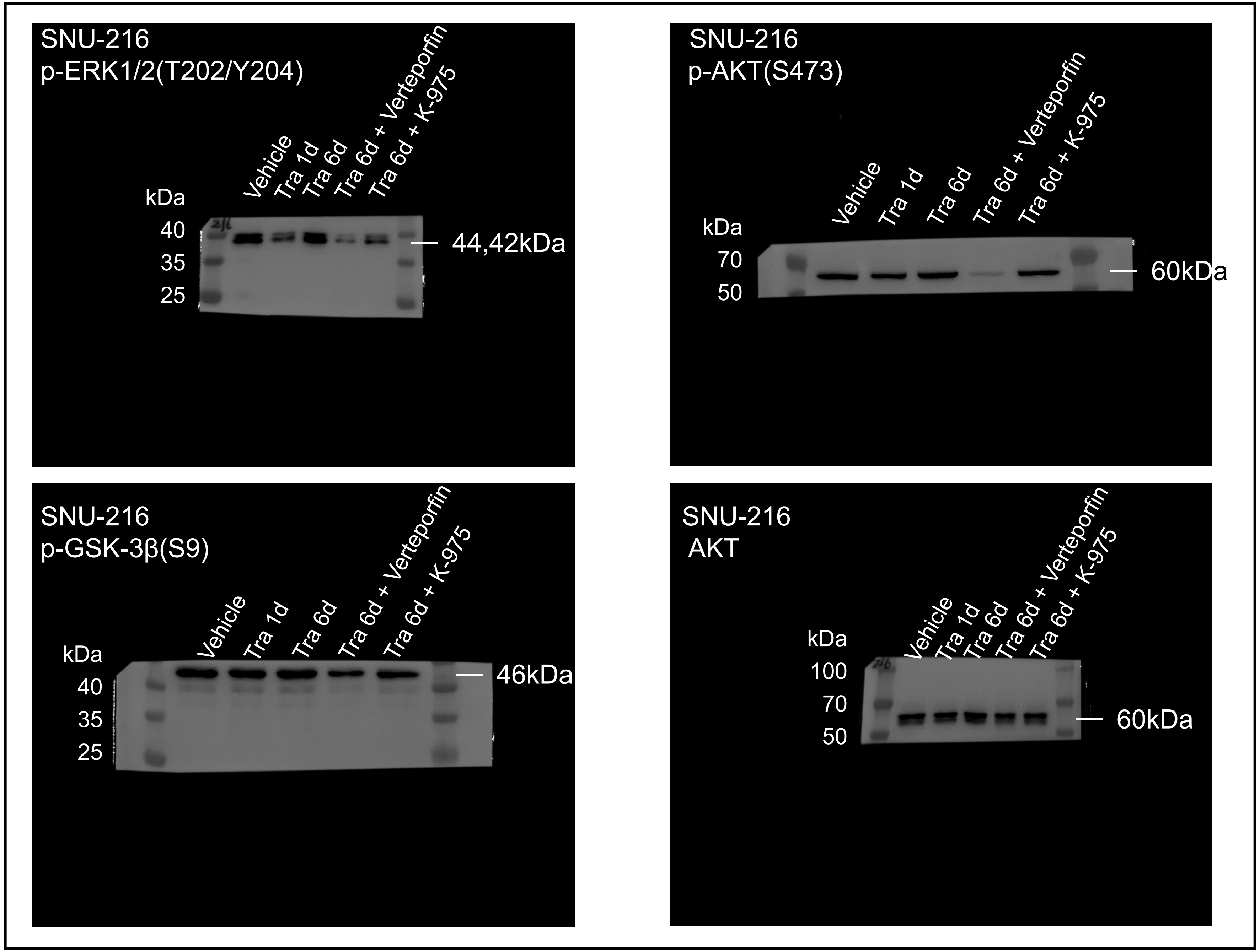
SNU-216
p-ERK1/2(T202/Y204)
Vehicle
Tra 1d
Tra 6d
Tra 6d + Verteporfin
Tra 6d + K-975
kDa
40
35
25
44,42kDa
SNU-216
p-AKT(S473)
Vehicle
Tra 1d
Tra 6d
Tra 6d + Verteporfin
Tra 6d + K-975
kDa
70
50
60kDa
SNU-216
p-GSK-3β(S9)
Vehicle
Tra 1d
Tra 6d
Tra 6d + Verteporfin
Tra 6d + K-975
kDa
40
35
25
46kDa
SNU-216
AKT
Vehicle
Tra 1d
Tra 6d
Tra 6d + Verteporfin
Tra 6d + K-975
kDa
100
70
50
60kDa

Fig. 4d

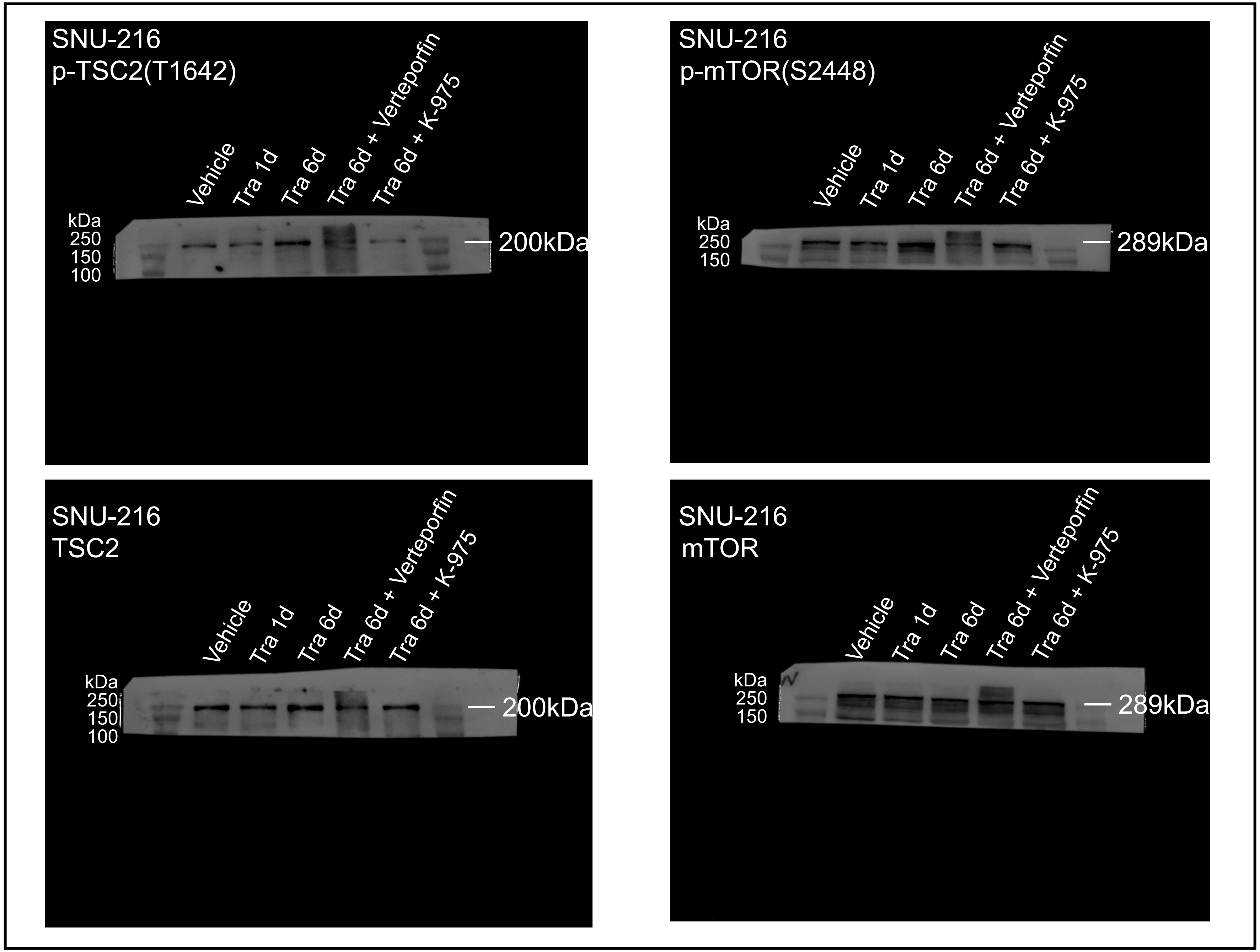

SNU-216
p-TSC2(T1642)
Vehicle
Tra 1d
Tra 6d
Tra 6d + Verteporfin
Tra 6d + K-975
kDa
250
150
100
200kDa
SNU-216
p-mTOR(S2448)
Vehicle
Tra 1d
Tra 6d
Tra 6d + Verteporfin
Tra 6d + K-975
kDa
250
150
289kDa
SNU-216
TSC2
Vehicle
Tra 1d
Tra 6d
Tra 6d + Verteporfin
Tra 6d + K-975
kDa
250
150
100
200kDa
SNU-216
mTOR
Vehicle
Tra 1d
Tra 6d
Tra 6d + Verteporfin
Tra 6d + K-975
kDa
250
150
289kDa

Fig. 4d

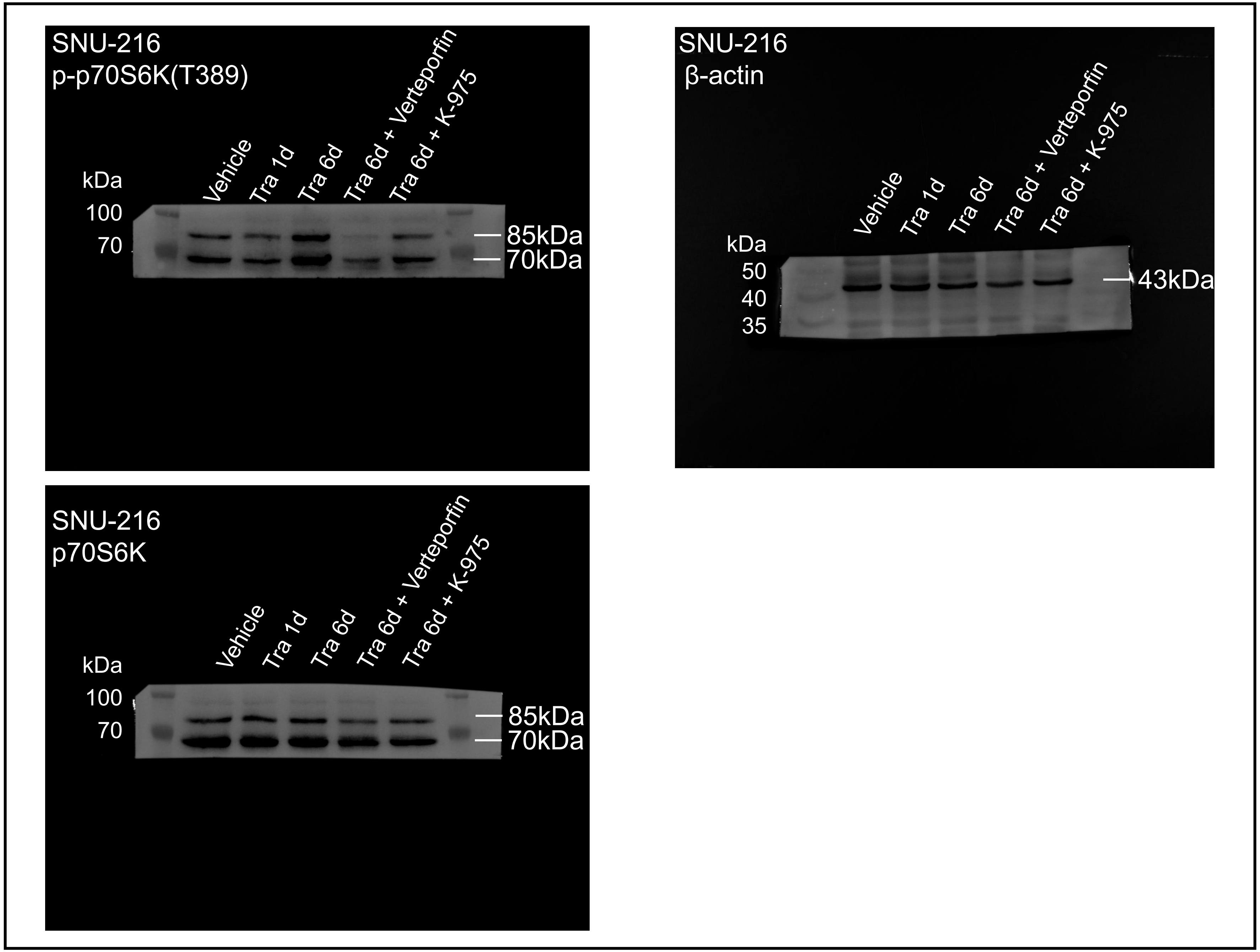
SNU-216
p-p70S6K(T389)
Vehicle
Tra 1d
Tra 6d
Tra 6d + Verteporfin
Tra 6d + K-975
kDa
100
70
85kDa
70kDa
SNU-216
β-actin
Vehicle
Tra 1d
Tra 6d
Tra 6d + Verteporfin
Tra 6d + K-975
kDa
50
40
35
43kDa
SNU-216
p70S6K
Vehicle
Tra 1d
Tra 6d
Tra 6d + Verteporfin
Tra 6d + K-975
kDa
100
70
85kDa
70kDa

Fig. 5g

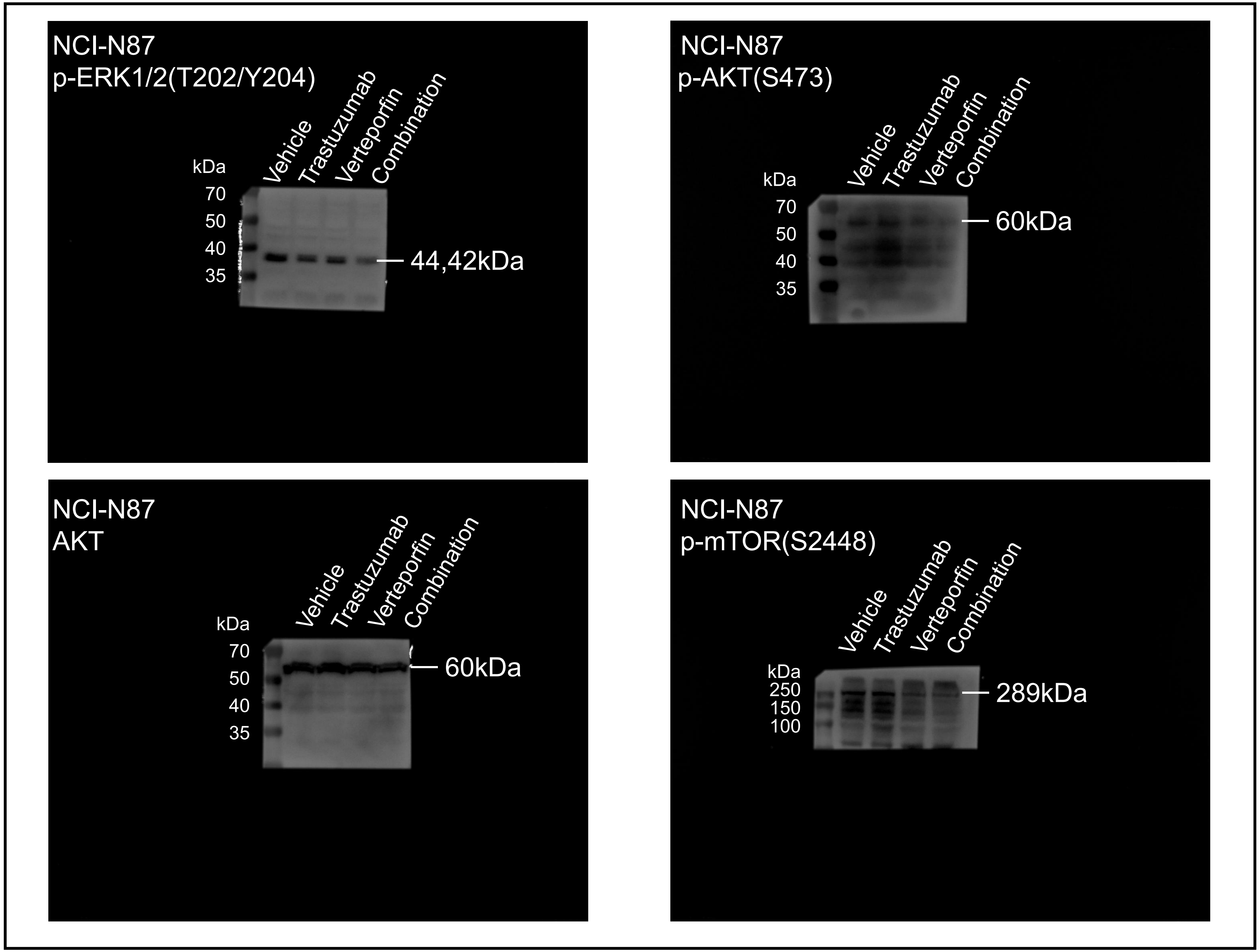
NCI-N87
p-ERK1/2(T202/Y204)
Vehicle
Trastuzumab
Verteporfin
Combination
kDa
70
50
40
35
44,42kDa
NCI-N87
p-AKT(S473)
Vehicle
Trastuzumab
Verteporfin
Combination
kDa
70
50
40
35
60kDa
NCI-N87
AKT
Vehicle
Trastuzumab
Verteporfin
Combination
kDa
70
50
40
35
60kDa
NCI-N87
p-mTOR(S2448)
Vehicle
Trastuzumab
Verteporfin
Combination
kDa
250
150
100
289kDa

Fig. 5g

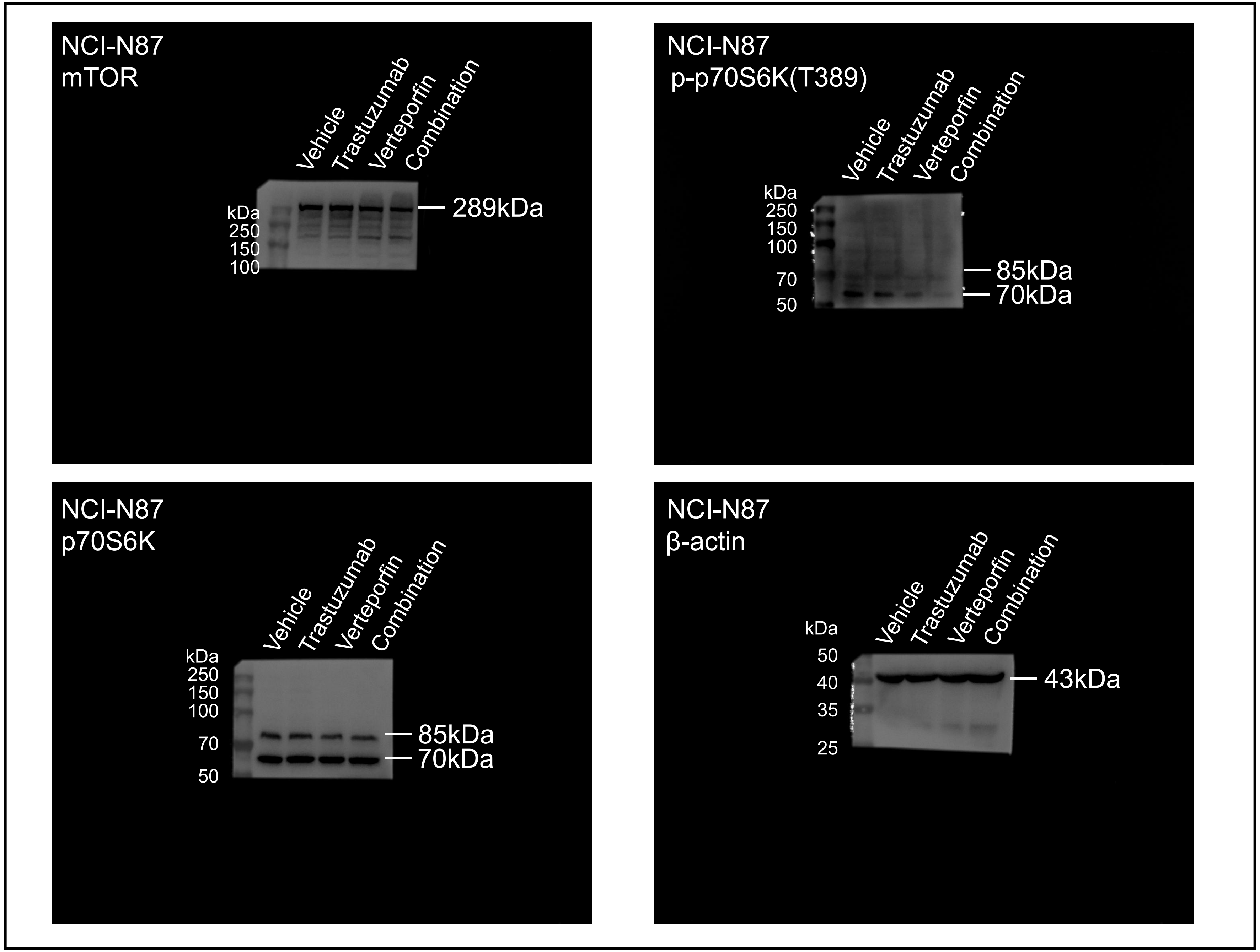
NCI-N87
mTOR
Vehicle
Trastuzumab
Verteporfin
Combination
kDa
250
150
100
289kDa
NCI-N87
p-p70S6K(T389)
Vehicle
Trastuzumab
Verteporfin
Combination
kDa
250
150
100
70
50
85kDa
70kDa
NCI-N87
p70S6K
Vehicle
Trastuzumab
Verteporfin
Combination
kDa
250
150
100
70
50
85kDa
70kDa
NCI-N87
β-actin
Vehicle
Trastuzumab
Verteporfin
Combination
kDa
50
40
35
25
43kDa

Fig. 5g

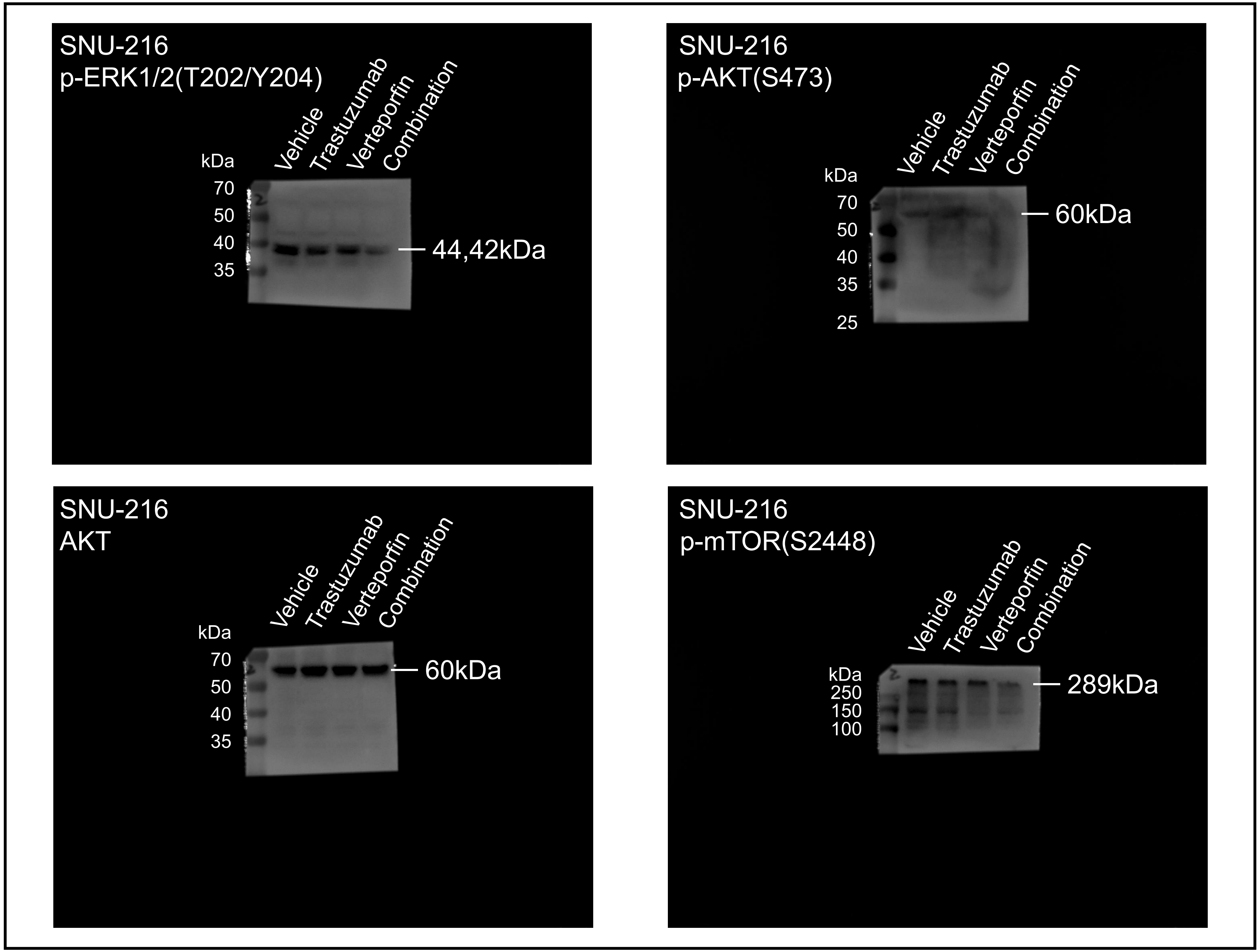
SNU-216
p-ERK1/2(T202/Y204)
Vehicle
Trastuzumab
Verteporfin
Combination
kDa
70
50
40
35
44,42kDa
SNU-216
p-AKT(S473)
Vehicle
Trastuzumab
Verteporfin
Combination
kDa
70
50
40
35
25
60kDa
SNU-216
AKT
Vehicle
Trastuzumab
Verteporfin
Combination
kDa
70
50
40
35
60kDa
SNU-216
p-mTOR(S2448)
Vehicle
Trastuzumab
Verteporfin
Combination
kDa
250
150
100
289kDa

Fig. 5g

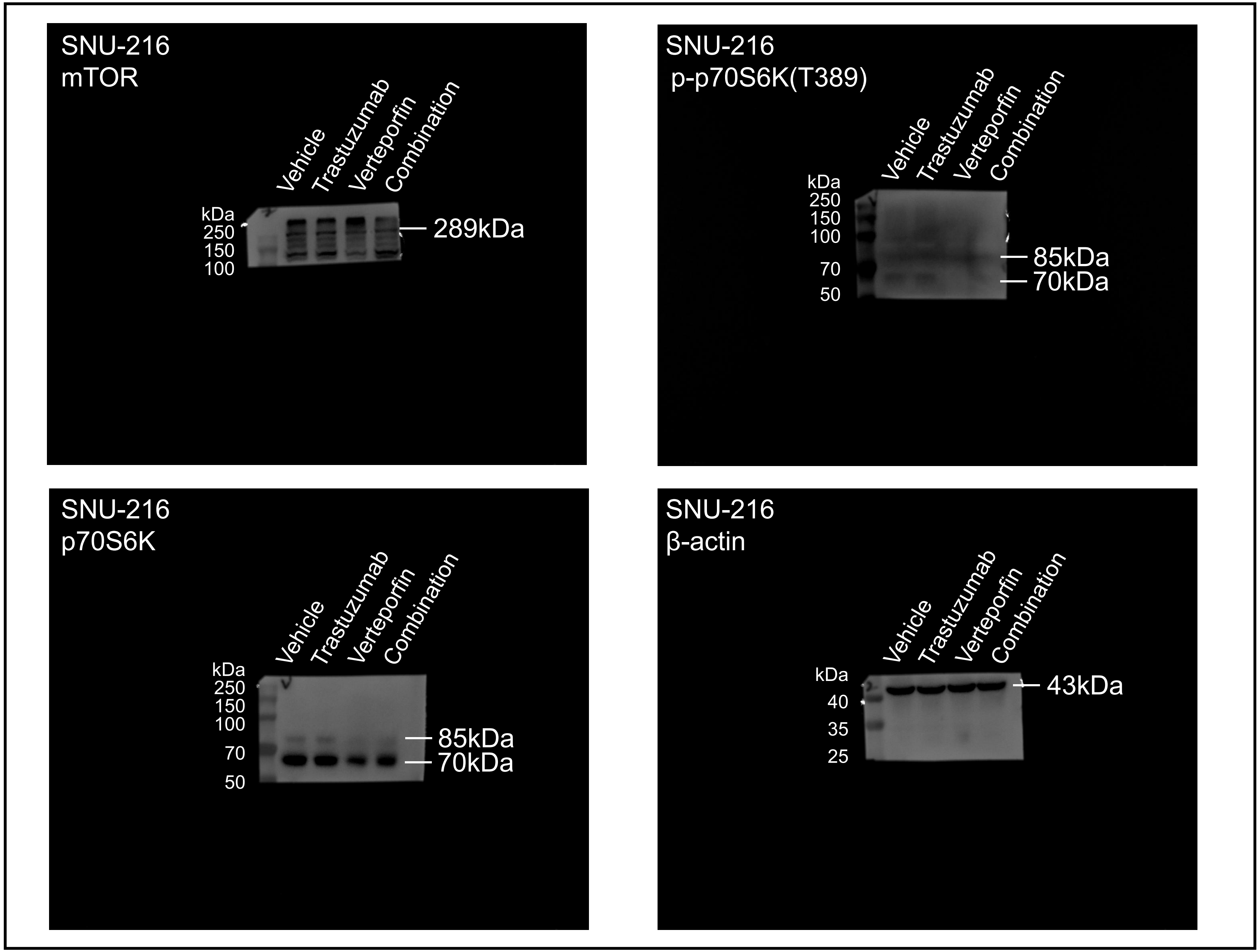
SNU-216
mTOR
Vehicle
Trastuzumab
Verteporfin
Combination
kDa
250
150
100
289kDa
SNU-216
p-p70S6K(T389)
Vehicle
Trastuzumab
Verteporfin
Combination
kDa
250
150
100
70
50
85kDa
70kDa
SNU-216
p70S6K
Vehicle
Trastuzumab
Verteporfin
Combination
kDa
250
150
100
70
50
85kDa
70kDa
SNU-216
β-actin
Vehicle
Trastuzumab
Verteporfin
Combination
kDa
40
35
25
43kDa

Fig. 5h

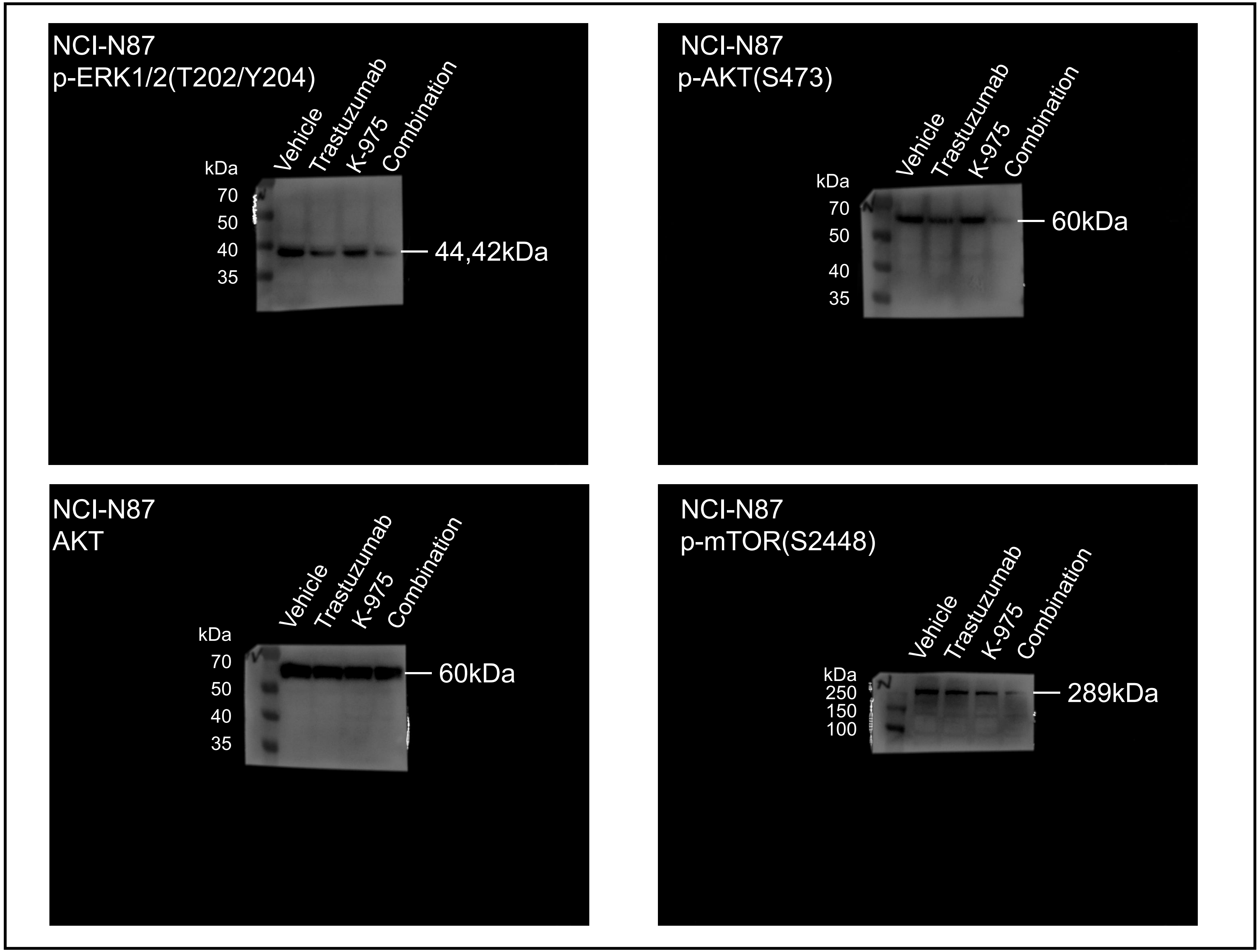
NCI-N87
p-ERK1/2(T202/Y204)
Vehicle
Trastuzumab
K-975
Combination
kDa
70
50
40
35
44,42kDa
NCI-N87
p-AKT(S473)
Vehicle
Trastuzumab
K-975
Combination
kDa
70
50
40
35
60kDa
NCI-N87
AKT
Vehicle
Trastuzumab
K-975
Combination
kDa
70
50
40
35
60kDa
NCI-N87
p-mTOR(S2448)
Vehicle
Trastuzumab
K-975
Combination
kDa
250
150
100
289kDa

Fig. 5h

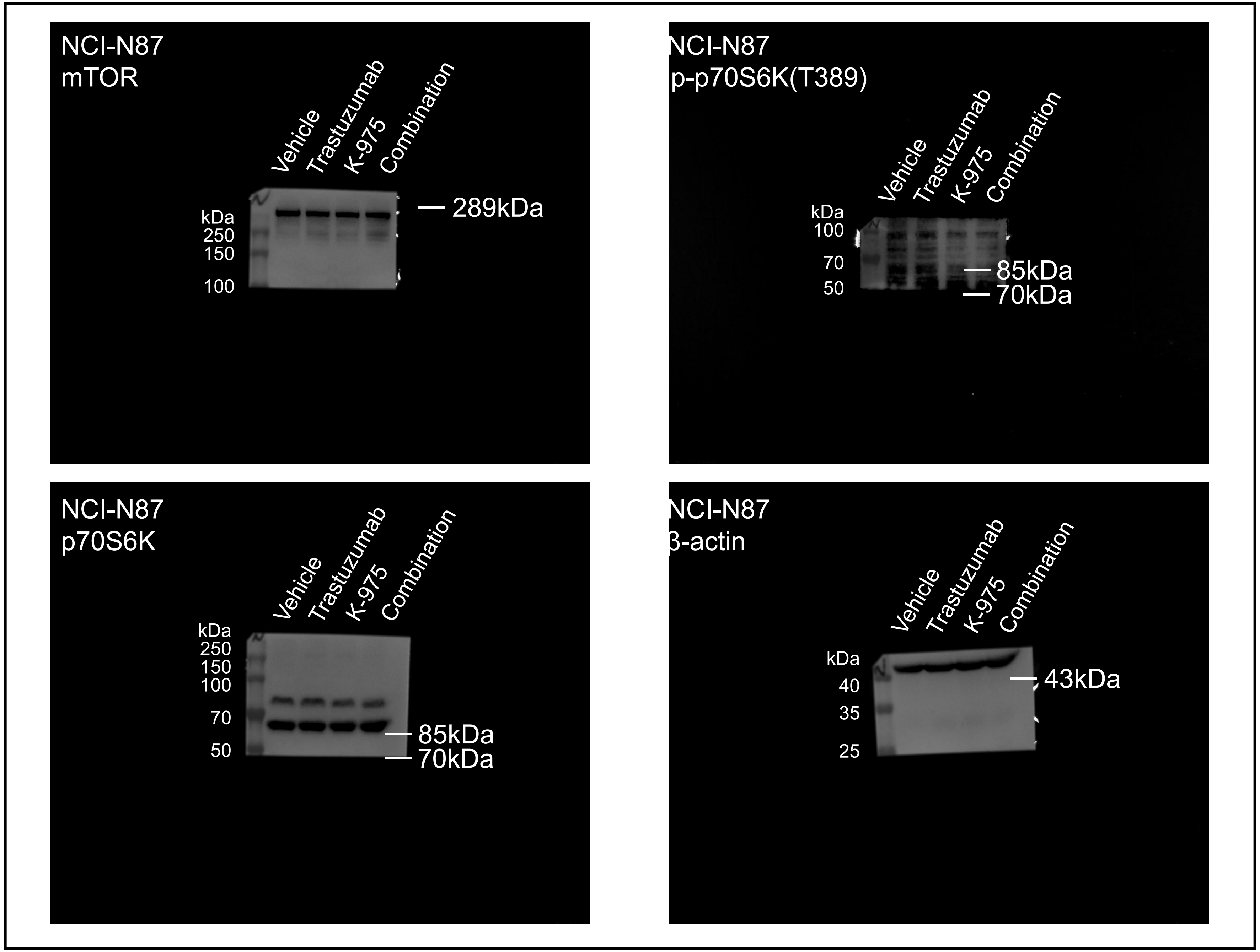
NCI-N87
mTOR
Vehicle
Trastuzumab
K-975
Combination
kDa
250
150
100
289kDa
NCI-N87
p-p70S6K(T389)
Vehicle
Trastuzumab
K-975
Combination
kDa
100
70
50
85kDa
70kDa
NCI-N87
p70S6K
Vehicle
Trastuzumab
K-975
Combination
kDa
250
150
100
70
50
85kDa
70kDa
NCI-N87
β-actin
Vehicle
Trastuzumab
K-975
Combination
kDa
40
35
25
43kDa

Fig. 5h

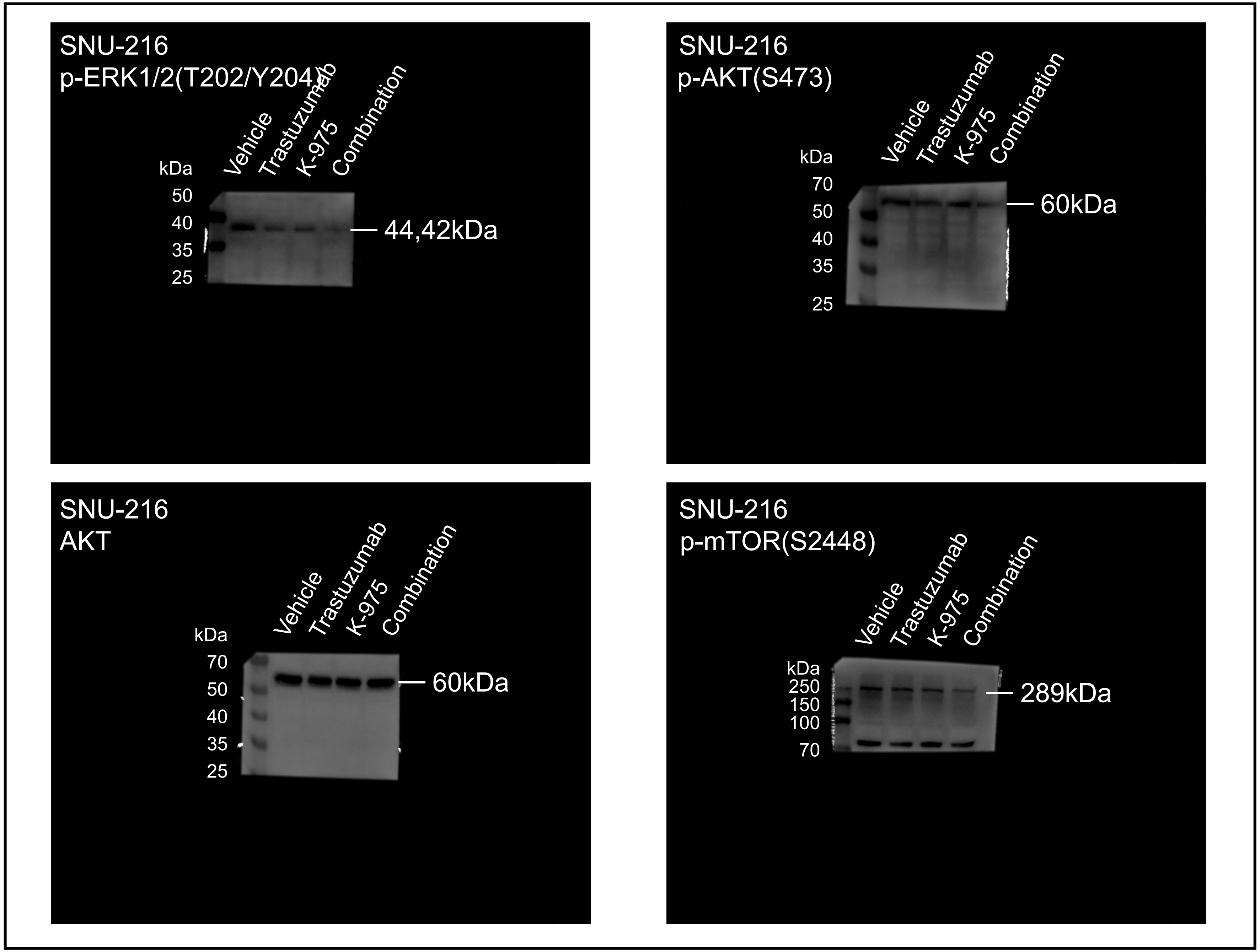
SNU-216
p-ERK1/2(T202/Y204)
Vehicle
Trastuzumab
K-975
Combination
kDa
50
40
35
25
44,42kDa
SNU-216
p-AKT(S473)
Vehicle
Trastuzumab
K-975
Combination
kDa
70
50
40
35
25
60kDa
SNU-216
AKT
Vehicle
Trastuzumab
K-975
Combination
kDa
70
50
40
35
25
60kDa
SNU-216
p-mTOR(S2448)
Vehicle
Trastuzumab
K-975
Combination
kDa
250
150
100
70
289kDa

Fig. 5h

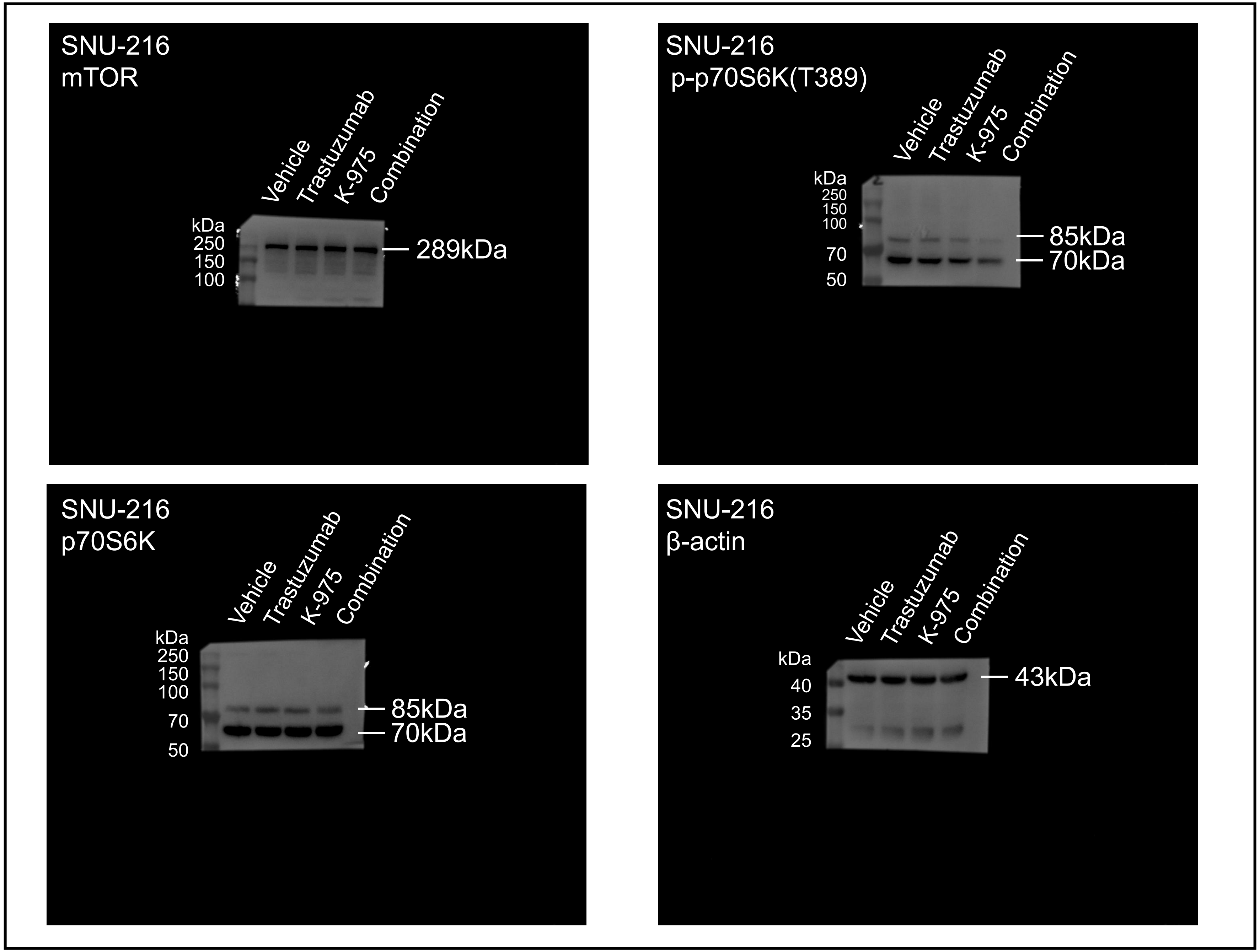
SNU-216
mTOR
Vehicle
Trastuzumab
K-975
Combination
kDa
250
150
100
289kDa
SNU-216
p-p70S6K(T389)
Vehicle
Trastuzumab
K-975
Combination
kDa
250
150
100
70
50
85kDa
70kDa
SNU-216
p70S6K
Vehicle
Trastuzumab
K-975
Combination
kDa
250
150
100
70
50
85kDa
70kDa
SNU-216
β-actin
Vehicle
Trastuzumab
K-975
Combination
kDa
40
35
25
43kDa
